# Dual-Targeted Gold Nanoprism for Recognition of Early Apoptosis, Dual-Model Imaging and Precise Cancer Photothermal Therapy

**DOI:** 10.7150/thno.34755

**Published:** 2019-07-28

**Authors:** Weiwei Zhang, Xiaoyuan Ding, Hao Cheng, Chenyang Yin, Jing Yan, Zhipeng Mou, Weiyun Wang, Danxi Cui, Cundong Fan, Dongdong Sun

**Affiliations:** 1 School of Life Sciences, Anhui Agricultural University, Hefei, 230036, China; 2 Key Lab of Cerebral Microcirculation in Universities of Shandong, Shandong First Medical University & Shandong Academy of Medical Sciences, Taian, Shandong, 271000, China

**Keywords:** Gold nanoprisms, aggregation-induced emission, apoptosis, reactive oxygen species, photothermal therapy

## Abstract

Photothermal therapy as novel strategy to convert near-infrared (NIR) light into heat for treatment cancers has attracted great attention and been widely studied. However, side effects and low efficiency remain the main challenge of precise cancer photothermal therapy.

**Methods:** In this study, we have successfully fabricated and characterized the dual-targeted gold nanoprisms, whereby bare gold nanoprisms (Au NPR) were conjugated to a phenanthroline derivatives-functionalized tetraphenylethene (TPE) and further stabilized with target peptide aptamers via Au-S bonds (Au-Apt-TPE). Then, the remaining nitrogen atoms of the Au-Apt-TPE could effectively chelate with Zn^2+^ ions (Au-Apt-TPE@Zn) for monitoring early stage apoptotic cells.

**Results:** The as-synthesized Au-Apt-TPE@Zn exhibited good monodispersity, size stability and consistent spectral characteristics. TPE synthesized here showed aggregation-induced emission (AIE) characteristics, and zinc conjunction (TPE@Zn) endowed Au-Apt-TPE@Zn with the cell membrane-targeted ability to selectively recognize the membranes of early stage apoptotic cells but not respond to healthy cells, which provided valuable diagnosis information on therapeutic efficacy. Au-Apt-TPE@Zn achieved specifically nuclear-targeted ability by surface decoration of AS1411 DNA aptamer. Au-Apt-TPE@Zn under NIR irradiation showed effective photothermal therapy against SGC-7901 human gastric carcinoma cells growth *in vitro* by inducing apoptosis through triggering reactive oxygen species (ROS) overproduction and regulating multiple signal crosstalk. *In vivo* studies revealed that Au-Apt-TPE@Zn under NIR irradiation showed deep penetration and dual-model imaging application (cancer-targeted fluorescence imaging and light-up photoacoustic imaging). Au-Apt-TPE@Zn under NIR irradiation also displayed strong photothermal therapy against gastric carcinoma xenograft growth *in vivo* by induction of apoptosis. Importantly, analysis of histopathology, hematotoxicity and immunocytotoxicity indicated that Au-Apt-TPE@Zn had less side effect and high biocompatibility.

**Conclusions:** Our findings validated the design of using Au nanoprism with AIE materials and dual-targeted decoration could be an effective strategy in recognition of early apoptosis, dual-model imaging and precise cancer photothermal therapy.

## Introduction

Gastric carcinoma as the most commonly diagnosed human cancer represents the most common cause of cancer death worldwide 1-3. Owing to its high malignancy, invasiveness and chemoresistance, traditional surgical resection, chemotherapy and radiotherapy were not appropriate for treatment of human gastric carcinoma. New therapeutics, such as cancer-targeted drug design, photodynamic therapy (PDT) and photothermal therapy (PTT), are urgently needed for cancer treatment 2, 4, 5.

Photothermal therapy (PTT) depends on photothermal agents that convert near-infrared (NIR) light into cytotoxic heat for tumor treatment. PTT as a new strategy exhibits low side effect, and minimal autofluorescence but deep penetration under NIR irradiation, which has attracted much attention and been widely studied 6, 7. Gold nano-materials as a promising PTT agent has been well developed and several gold nano-materials-based PTT have reached clinical trials 4, 8, 9. Mitochondrial respiratory chain was the main source of cytotoxic reactive oxygen species (ROS), and dysfunction of mitochondria and antioxidases may cause ROS accumulation. PTT under NIR irradiation may cause ROS overproduction, such as hydroxyl radicals 10, hydrogen peroxide 11, superoxide 12 and singlet oxygen (^1^O_2_) 13. Overproduced ROS can damage DNA, proteins and lipids, and eventually cause cancer cells apoptosis, which was accepted as the main anticancer mechanism of PTT 8, 14. Hence, design of novel photothermal agents with high specificity, high efficiency, deep penetration and low side effect has become the research focus nowadays 15-17.

Plasmonic materials with different compositions, structures, shapes, and surface coatings can be used for PTT under NIR irradiation 18, 19. PTT with unique advantages has been widely applied for drug delivery, and photoacoustic (PA) imaging, as well as biosensing 16, 17, 20, 21. Nanoprisms can act as potential efficient nanoheaters for PA imaging and ablation of cancer cells *in vitro* and* in vivo* 17, 22, 23. Gold nanoprisms (Au NPRs) was accepted as good drug carrier and can be modified with bio-related molecules to enhance cancer-targeted specificity 24. Multifunctional nano-composites have been well developed and attracted significant attention due to their unique structures and excellent performance 25, 26. Conventional photosensitizers may be quenched by aggregation, and design of materials with aggregation-induced emission (AIE) characteristics has become the research focus 27, 28. Tetraphenylethene (TPE) as a conjugated helical molecule shows highly emissive in the aggregate state, where the restriction of the intramolecular rotation of the phenyl substituent can be inhibited by non-radiative decay pathways 28-31. Hence, nanoprisms decorated with AIE-based materials are alternative tools for cancer diagnosis and treatment, which overcomes the limitations of conventional photosensitizers that exhibit aggregation-induced quenching effects.

Cancer-targeted drug design with high efficiency and low side effect has been widely studied and applied to precise drug delivery and cancer therapy. Apoptosis can provide valuable diagnosis information on therapeutic efficacy, and many efforts had been made to monitor the intracellular apoptotic process 32. Phosphatidylserine (PS) usually transfer to the outside of cell membrane in response to apoptotic stress and was commonly used as a marker for early stage apoptosis. The fluorescent dye-labeled annexin V was the most widely used probe for detecting PS expression. However, annexin V has a short shelf life and needs extracellular Ca^2+^. Hence, cell membrane-targeted drugs design to selectively recognize the membranes of early stage apoptotic cells is of great significance. Recently, fluorescent dyes-conjugated zinc-dipicolylamine complexes can act as a substitution of annexin V probe to detect early stage apoptosis 32, 33. Herein, tetraphenylethene (TPE-SH) a novel AIE materials was synthesized and modified with zinc (Zn^2+^), which endowed TPE@Zn with the ability for cell membrane-targeted fluorescence imaging and selective recognition of early stage apoptosis. Meanwhile, a nucleolin-targeted DNA aptamers (Apt) AS1411 DNA was also modified to the surface of nanoprisms, which achieved dual-targeted design for fluorescence imaging and precise cancer PTT 34, 35.

In the present study, a multifunctional gold nanoprism (Au-Apt-TPE@Zn) was obtained by surface decoration of DNA aptamer (Apt) and Zinc-tetraphenylethene (TPE@Zn). Our results showed Au-Apt-TPE@Zn selectively recognized the membranes of early stage apoptotic and achieved nuclear-targeted ability. Au-Apt-TPE@Zn under NIR irradiation showed strong photothermal therapy against SGC-7901 human gastric carcinoma cells growth *in vitro* and *in vivo.* Au-Apt-TPE@Zn *in vivo* under NIR irradiation showed deep penetration and cancer-targeted fluorescence imaging and light-up photoacoustic imaging properities, but exhibited less side effect and high biocompatibility. Our findings validated the design of using Au nanoprism with AIE materials and dual-targeted decoration could be an effective strategy in recognition of early apoptosis, dual-model imaging and precise cancer photothermal therapy.

## Experimental Section

### Materials

Sodium citrate (99.9%), HAuCl_4_·3H_2_O (≥99.9%), polyvinylpyrrolidone (PVP, wt 40,000), NaOH (≥98.0%), and HCl (37%) were purchased from Sigma-Aldrich Chemical Co. (USA). The AS1411 DNA aptamer with a disulfide modification was synthesized by TaKaRa (Dalian, China). Hiazolyl blue tetrazolium bromide (MTT), Propidium Iodide (PI), 1,1'-dioctadecyl-3,3,3',3'-tetramethylindocarbocyanine (DiI), Calceinand Alexa Fluor 488 annexin V were all purchased from Sigma-Aldrich Chemical. All aqueous solutions were prepared in doubly distilled water. All other reagents were the best commercially available.

### Synthesis of Au NPRs

1 mL of HAuCl_4_·4H_2_O (1%) was dissolved in 25 mL of Milli-Q water (pH=3.5), and 50 mg of PVP was added into the solution. The obtained solution was poured into a three-necked flask (150 mL) and was irradiated by microwave (MW) for 5 min with mechanical stirring. Then 1.5 mL of sodium citrate solution (1%) was quickly added into the solution and was irradiated by MW for 3 min. The solution was collected by centrifugation at 10000 rpm for 5 min as previously reported 36-38.

### Assembly of Au-Apt NPRs

The disulfide bond of the aptamers was cleaved by tris (2-carboxyethyl) phosphine. After 30 min, the aptamer (100 μL, 100 μM) solution was added to 10 mL of a 5 nM solution of Au NPRs and incubated to form Au-Apt NPRs for 24 hours (Apt/AuNPRs=200:1, mol ratio). The next day, we salted the mixture solution with 2.5mL of a 500 mM solution of NaCl twice and separated by 4 h 34.

### Assembly of Au-TPE NPRs

The solution of Au NPRs was dispersed in Milli-Q H_2_O (2 mL). The solution of Au NPRs was added to a solution of 6.86 mg TPE (THF, 10 mL) under the protection of nitrogen (TPE/AuNPRs=200:1, mol ratio). TPE was prepared separately in our laboratory. Then the reaction solution was protected from light and preceded for 48 h. Then, the resultant Au-TPE solution was collected and washed for three times (10 min, 10000 rpm) to remove the excessive TPE molecules. The assembled Au-TPE was then re-dispersed in water (10 mL).

### Assembly of Au-Apt-TPE and Au-Apt-TPE@Zn NPRs

The solution of Au-Apt was collected and washed three times (10 min, 10000 rpm) to remove the excessive DNA and NaCl. Then the precipitate (Au-Apt) were dispersed in Milli-Q H_2_O (2 mL) 39. Next, the solution of Au-Apt was added to a solution of 6.86 mg TPE (THF, 10 mL) under the protection of nitrogen. TPE was prepared separately in this laboratory. Then the reaction solution was protected from light and preceded for 48 hours. Then, the resultant Au-Apt-TPE solution was collected and washed for three times (10 min, 10000 rpm) to remove the excessive the TPE molecules. The resulting Au-Apt-TPE was re-dispersed in water (10 mL). An aqueous solution of zinc perchlorate hexahydrate (10 mL, 10 mmol) were added to Au-Apt-TPENPRs solution (10 mL) and stirred for 2 h at room temperature. The resultant Au-Apt-TPE@Zn NPRs solution was centrifuged for 10 min at 10000 rpm to remove the excessive zinc perchlorate hexahydrate 33. The final Au-Apt-TPE NPRs were re-dispersed in water (10 mL). The synthetic route of TPE@Zn could be found in the **Figure** S1.

### Characterization of Au-Apt-TPE@Zn NPRs

The UV-visible absorption spectra were registered using ultraviolet-visible spectroscopy (UV-vis, S-3100 photodiode array, Scinco Co., Korea). Fourier transform infrared spectroscopy was performed on a FT-IR spectrometer (Nicolette is50, America, Thermo Fisher Scientific). Morphology of the Au NPRs and Au-Apt-TPE@Zn NPRs were examined by a transmission electron microscope (TEM, HT7700, Tokyo Japan, Hitachi). The dynamic light scattering (DLS) and zeta potentials measurements were used for characterization of Au-Apt-TPE NPRs optical properties and sizes on a Brookhaven Zeta PALS instrument. Nanoparticle suspensions (30 µg/mL) were illuminated with an 808 nm NIR for 30 min. The temperature was detected through an infrared camera with an accuracy of 0.1℃ 27, 33, 40.

### Cell culture and *in vitro* anticancer effects

SGC-7901 human gastric carcinoma cells were cultured in RPMI with 10% fetal bovine serum (FBS) at 37℃ and 5% CO_2_ 41.SGC-7901 cells seeded in 96-well plates were treated with 2.5-30 µg/mL Au-Apt-TPE@Zn for 12 h, and cells were co-treated with or without 808 nm NIR irradiation (200 mW/cm^2^) for 20 min. Cell viability was detected by MTT assay. For different NIR treatment, SGC-7901 cells seeded in 48-well plate (2.0×10^4^ cells/well) were incubated with 30 µg/mL Au-Apt-TPE@Zn for 12 h and co-treated with 808 nm NIR (40 mW/cm^2^, 80 mW/cm^2^, 160 mW/cm^2^, 320 mW/cm^2^ and 500 mW/cm^2^). Cell viability at different time (0, 5, 10, 20 and 30 min) were measured by Calcine AM/PIco-staining. (PI, Ex = 535 nm, and Em = 615 nm. annexin V-FITC, Ex = 488 nm, and Em = 525 nm).

### Detection of early stage apoptosis

SGC-7901 cells (2.0×10^4^ cells/well) seeded in 48-well plate were treated with 250 μM H_2_O_2_ for 6 h. Then cells were gently washed with fresh medium and co-stained with 5 µg/mL of Au-Apt-TPE or Au-Apt-TPE@Zn NPRs for 2 h. Then cells were co-stained with 10 µM DiI and exposed to NIR irradiation. Fluorescence images were obtained using a fluorescence microscope. For DiI, Ex=549 nm, and Em=565 nm.

### *In vitro* cellular uptake and real-time fluorescent imaging

SGC-7901 cells (10^5^ cells/well) seeded in 6-well plate were treated with 30 µg/mL Au-Apt-TPE@Zn for 12 h. Then, cells were harvested, washed with PBS and fixed with 2% paraformaldehyde and 2.5% glutaraldehyde for 12 h. Then, the cells were air-dried on 200-mesh copper grids and detected by with TEM 8, 42. For real-time fluorescent imaging, SGC-7901 cells were seeded in 48 well plates at 37 °C and grown for 24 h. cells were treated with 250 μM H_2_O_2_ for 6 h followed by staining with Au-Apt-TPE (5 µg/mL) and Au-Apt-TPE@Zn NPRs (5 µg/mL). The real-time fluorescent images were imaged at different times by a fluorescence microscope. The fluorescent images were taken at different times with Ex=372 nm, and Em=450 nm.

### Measurement ROS generation

For the detection of ROS generation, SGC-7901 cells (10^5^cells/well) seeded in 6-well plate were treated with 30 µg/mL Au-Apt-TPE@Zn for 12 h, and cells were co-treated with or without NIR irradiation. Then, cells were incubated with 10 μM DCFH-DA probe at 37℃ for 20 min. Then, cells were washed with PBS and ROS generation was examined with an excitation wavelength and emissions wavelength at 488 and 525 nm, respectively 43.

### Western blot analysis

Cells after treatment with Au-Apt-TPE@Zn and/or NIR irradiation, total proteins were isolated and quantified by BCA kit. The protein expression was examined by western blot method as described before 43, 44.

### Real-time PCR

SGC-7901 cells after treatment with Au-Apt-TPE@Zn and/or NIR irradiation, real-time PCR was performed with a cDNA Synthesis Kit and FastStart Universal SYBR Green Master (Rox) using a StepOne real-time PCR system (Applied Biosystems, Thermo Fisher Scientific, Waltham, MA). The quantity of cDNA measured by real-time PCR was normalized by the abundance of 16S cDNA. All of the assays were repeated at least 3 times with similar results.

### *In vivo* anticancer effects

BALB/c male nude mice were purchased from the Model Animal Research Center of Nanjing University and bred in an axenic environment (SPF). Tumor models were established by subcutaneous injection of SGC-7901 cells (10^6^ cells in 100 µL) into the shoulder of the nude mice. When the volume of tumors reached 50 mm^3^, mice were randomly divided into four groups. PBS-treated group, NIR-treated group, Au-Apt-TPE@Zn-treated group, and Au-Apt-TPE@Zn + NIR-treated group. Mice were given 2 mg/kg Au-Apt-TPE@Zn by intravenous tail injections. After 24 h, tumor regions were exposed to 808 nm NIR (320 mW/cm^2^) for 20 min. Tumor volume was calculated 18 days after the NIR treatment using length × width^2^ ×0.5 45. Tumors and organs were collected for hematoxylin-eosin (H&E) staining 44. Cells apoptosis in tumor tissue was detected by TUNEL assay. All animal experiments were carried out according to the protocols approved by Anhui Agricultural University Laboratory Animal Center (Permit Number: SYXK 2016-007). For DAPI imaging, Ex = 358 nm, and Em = 461 nm. For TUNEL, Ex =450-500 nm, and Em = 515-565 nm.

### *In vivo* biodistribution

The photoacoustic (PA) signal intensity of Au-Apt-TPE@Zn NPRs with varied concentrations (1.25, 2.5, 5, 10, 15, 20 and 30 µg/mL) was determined with a commercial PA imaging system at 808 nm. Mice were administered intravenous tail injections with Au-Apt-TPE@Zn NPRs (2 mg/kg), and the PA signal of NPRs was detected at 0, 2, 6, 10, 14 and 20 h. The fluorescence images of the whole mice were detected at 2, 4, 6, 8, 10 and 12 h after injection using the IVIS Spectrum *in vivo* fluorescence imaging system (Caliper Perkin Elmer).

### MRI acquisition

The MRI images of the whole mice and tumor tissue with SGC-7901 xenograft were obtained on a 3.0-TMR scanner (General Electric, Milwaukee, WI, USA) and were examined at 0, 5, 10, 15, 20 and 25 days after different treatments. The T_2_-weighted images of the nude mice and tumor tissue were obtained 25 days after treatments 43.

### Safety evaluation of Au-Apt-TPE@Zn

To evaluate the toxicity of the Au-Apt-TPE@Zn NPRs and/or NIR treatments in vivo, the mice after treatment were sacrificed after 30 days, and the important organs (liver, spleen, kidneys, heart, and lungs) of mice were collected and stained with H&E for histopathological changes. Macrophage toxicity was detected by labeling Kupffer cells in liver using an anti-F4/80 antibody. Blood samples were also collected and hematological parameters, including uric acid, blood glucose, cholesterol, alanine aminotransferase, RBC number of red blood cells, WBC number of white blood cells, MCV mean corpuscular volume, HCT hematocrit, HGB hemoglobin, MCH mean corpuscular hemoglobin, AST aspartate aminotransferase, ALT alanine aminotransferase, were all measured by ACL-200 blood autoanalyzer (Beckman Coulter Corp., USA). Health nude mice were set as the blank 46, 47. *P<0.05 vs. blank, **P<0.01 vs. blank.

### Statistical analysis

All data and images were obtained from three independent experiments. Data were presented as mean ± SD. Statistical analysis was performed by SPSS 13.0 (SPSS, Inc.). Statistical analysis was conducted by one-way ANOVA followed by a Dunnett's or Tukey's post-hoc test. *P<0.05 vs. blank, **P<0.01 vs. blank.

## Results and Discussion

### Characterization of Au-Apt-TPE@Zn NPRs

**Figure 1:**
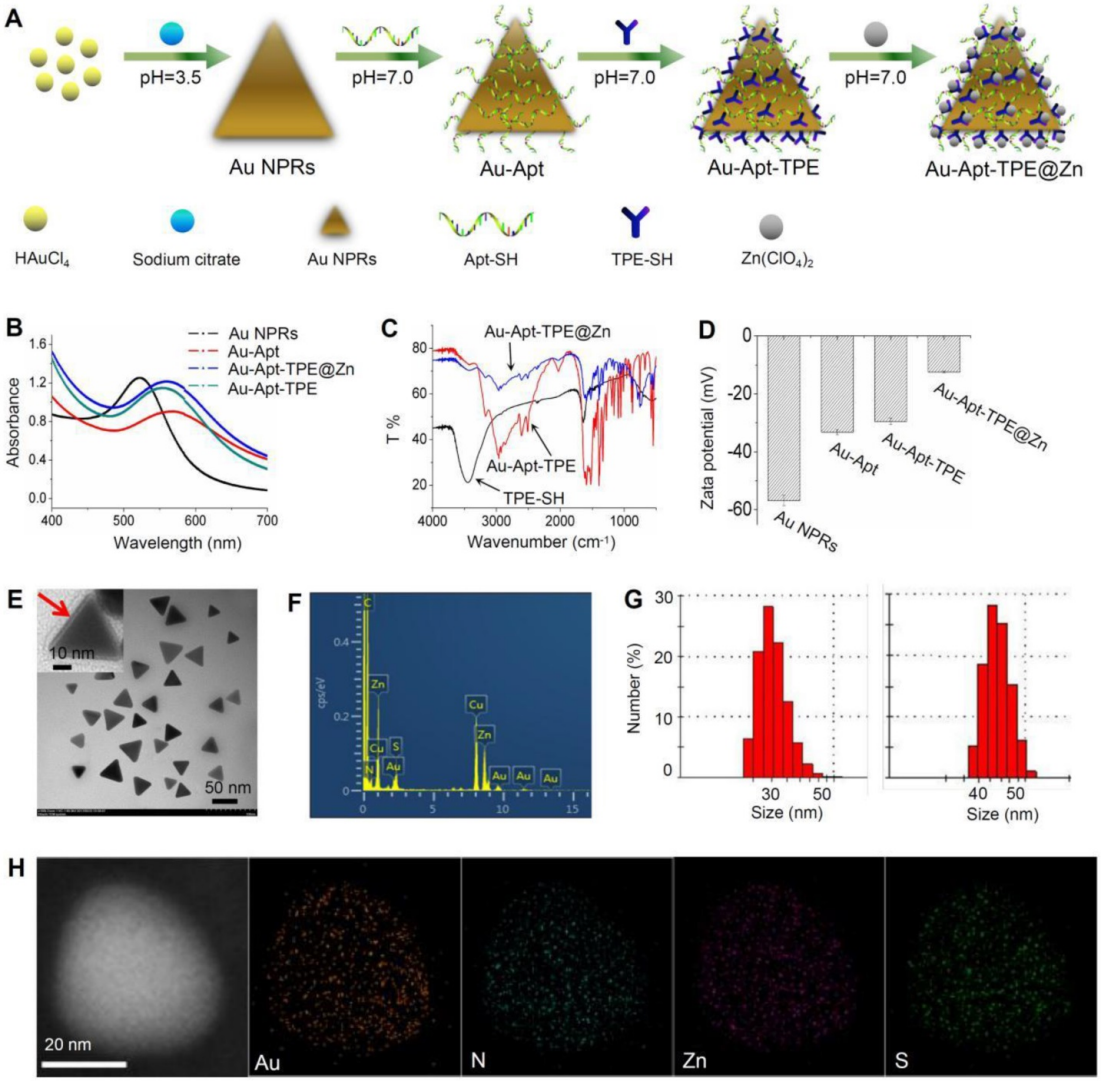
Synthesis and characterization of Au-Apt-TPE@Zn. (A) Synthesis illustration of Au-Apt-TPE@Zn. (B) Absorption spectra. (C) FT-IR spectrometer. (D) Zeta potential of Au NPRs, Au-Apt, Au-Apt-TPE and Au-Apt-TPE@Zn. (E) TEM images of Au-Apt-TPE@Zn. The red arrow indicates the coated Au-Apt-TPE@Zn. (F) TEM-EDS spectrum of Au-Apt-TPE@Zn NPRs. (G) Sizes of Au NPRs (left) and Au-Apt-TPE@Zn (right). (H) Element mapping of Au-Apt-TPE@Zn NPRs.

Bare Au NPR was synthesized using an improved microwave method according to previous literature 48. TPE-SH and target peptide aptamers were modified on the surface of Au NPR via Au-S bonds. Au-Apt-TPE could react with zinc perchlorate hexahydrate to directly provide the desired product of Au-Apt-TPE@Zn NPRs in an approximately quantitative yield (90%) (**Figure 1A**). The optical features of the gold nanostructures at different stages of the synthesis are shown in **Figure 1A** and **Figure S3**. The extinction spectrum of AuNPR (black line) exhibits a sharp plasmon resonance approximately at 537 nm, which is expected for AuNPR of this size (**Figure 1B**) 49. For the Au-Apt-TPE@Zn NPRs, the absorbance at 537 nm is red-shifted, and the emergence of a second peak in the NIR at 808 nm is apparent (**Figure S4**). Since light in the NIR region (e.g., 650-1000 nm) can penetrate soft tissues relatively well, the optical properties of Au-Apt-TPE@Zn are quite suited for applications such as contrast tools for PA or PTT agents 50. Their structures were also confirmed by nuclear magnetic resonance (NMR) (**Figure S2**), Fourier transform infrared (FTIR) spectroscopy (**Figure 1C**), showing the successful conjugation of aptamers on the surface of gold nanoprisms. The transmission electron microscopy (TEM) image showed that relatively homogeneous nanostructures had been successfully prepared (**Figure 1E**). The average edge length of the obtained nanostructures was 45±5.0 nm (**Figure 1F**). The zeta potential of TPE and Apt-capped Au-NPRs was -32.11±0.8 mV, while that of Au-Apt, Au-Apt-TPE and Au-Apt-TPE@Zn NPRs was -33.2±0.8 mV, -33.2±0.8 mV and -29.5±1.2 mV (**Figure 1D**). In addition to contrast difference, the hybrid Au-Apt-TPE@Zn NPRs structure can also be well observed by mapping of their element distribution (**Figure 1D**). Taken together, these results suggest TPE-SH and DNA aptamer were successfully decorated on the surface of gold nanoprisms.

### *In vitro* photothermal efficiency

**Figure 2:**
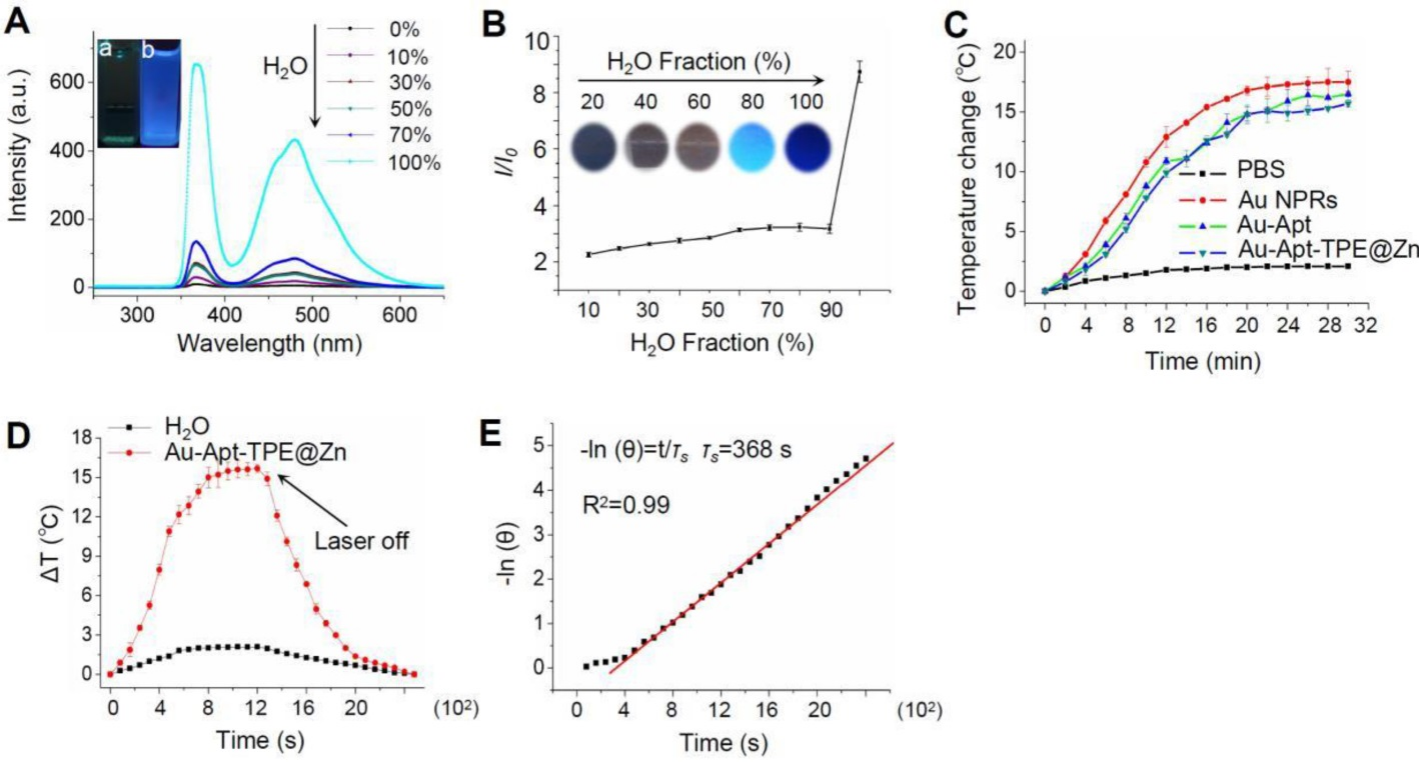
*In vitro* photothermal efficiency. (A) Fluorescence excitation spectra of Au-Apt-TPE@Zn in solvent containing different compositions of water/THF. Letter “a” indicates the100% THF, and letter “b” indicates 0% THF. (B) Plot of relative fluorescence intensity (I/I_0_) at 450 nm versus the solvent composition of the water/THF mixture of Au-Apt-TPE@Zn. (C) The photothermal responses of Au NPRs, Au-Apt, and Au-Apt-TPE@Zn. Different particles (30 µg/mL) were exposed to NIR irradiation (808 nm, 500 mW/cm^2^). PBS was set as a control. (D) Heating and cooling curves of Au-Apt-TPE@Zn NPRs (10 μg/ml) and Milli-Q water (at laser irradiation of 808 nm). The maximum temperature observed for the Au-Apt-TPE@Zn NPRs solution was much higher than that of PBS. (E) The linear regression between cooling period and -ln (θ) of driving force temperature.

The fluorescence excitation spectra of Au-Apt-TPE@Zn in different composition of water/THF were showed in the **Figure 2A**. The results showed that the fluorescence intensity of the solution became stronger and stronger with the increase proportion of water. The emission of Au-Apt-TPE@Zn at 450 nm in water and THF mixtures with different THF fractions were shown in **Figure 2B**. It shows that weaken fluorescence was observed in water and the fluorescence intensity increased significantly when the solution contained THF fraction (above 90%), showing a typical AIE phenomenon. In the incident radiation from appropriate wavelength excitation, NPRs through the absorption of light energy conversion produced significant localized heating, suggesting these specific particles could act as efficient “nano-heaters”. As expected, Au NPRs, Au-Apt, and Au-Apt-TPE@Zn exposed to 808 nm NIR irradiation acted as effective photothermal agents, which resulted in rapid warming (**Figure 2C**). The photothermal conversion efficacy of Au-Apt-TPE@Zn NPRs was determined following Chen's report 51. For Au-Apt-TPE@Zn NPRs at the concentration of 30 µg/mL, the *m*_H2O_ (m_D_) is 1.0 g and the *C*_H2O_ (C_D_) is 4.2 J/g/°C, the A (808 nm) is 0.3, ΔT_max_ is 16 °C (**Figure 2D**), I is 0.5 W, Q_s_ is 10.9 mW, *τ_s_* is determined to be 368 sec (**Figure 2E**), thus the photothermal conversion efficacy (*η*) of Au-Apt-TPE@ZnNPRscan be calculated to be 67.2%, which is higher than gold nanoshells (13%) 52, gold rod (21%) 53, gold hexapods (29.6%) 54, and gold nanocages (63%) 54 (Table S1).

### *In vitro* anticancer activity

**Figure 3:**
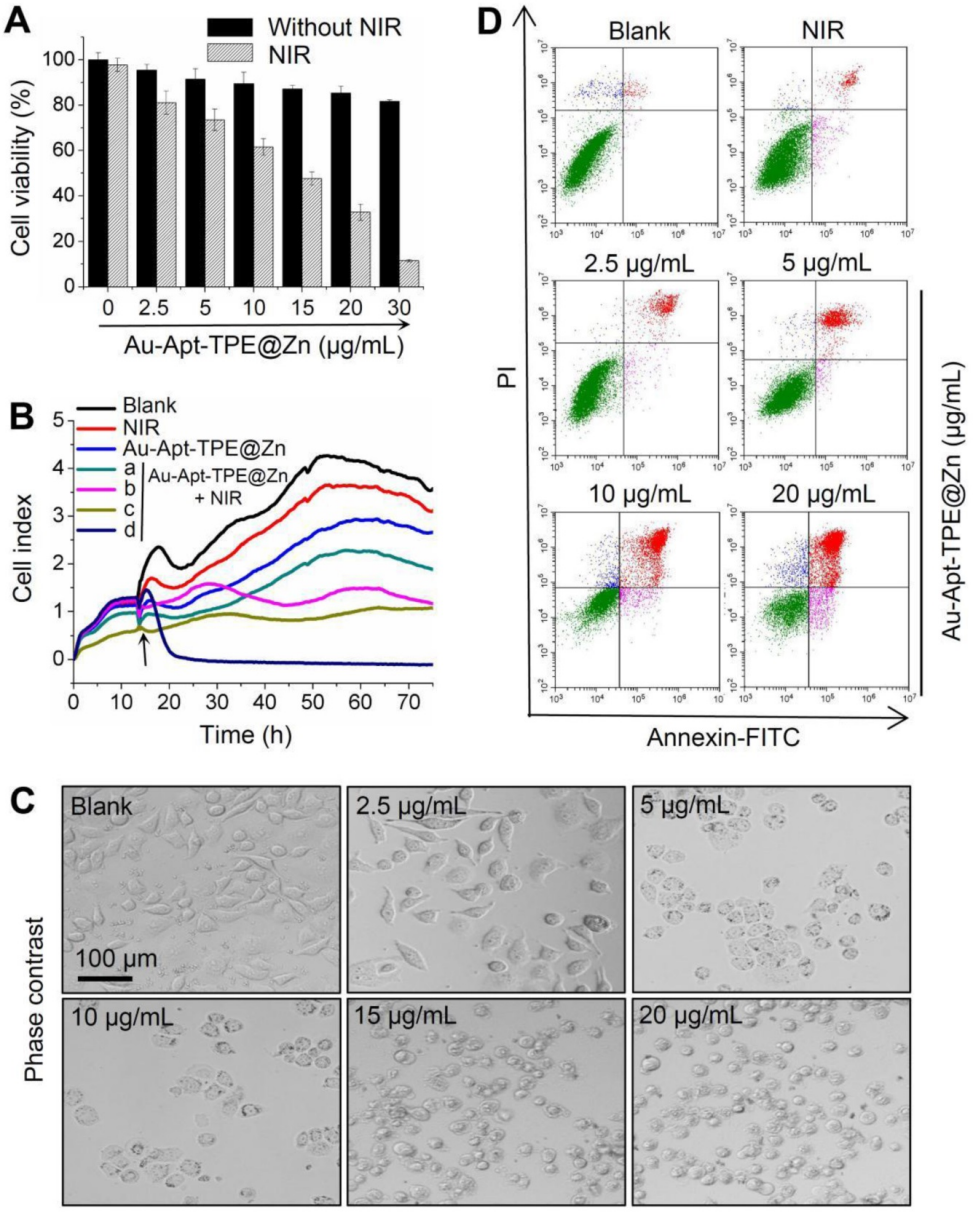
*In vitro* anticancer effects of Au-Apt-TPE@Zn. (A) Cytotoxicity of Au-Apt-TPE@Zn on SGC-7901 cells. SGC-7901 cells were treated with 2.5-30 µg/mL Au-Apt-TPE@Zn for 12 h and co-exposed to NIR irradiation (200 mW/cm^2^) for 20 min. Cell viability was detected by MTT assay. (B) Cell proliferation of SGC-7901 cells were treated with 2.5, 5, 10 and 20 µg/mL Au-Apt-TPE@Zn determined by RT-CES, respectively. (C) Observation of cells morphology. (D) Detection of cell apoptosis. Cells after treatment were stained by annexin V-FITC/PI and analyzed by flow cytometry.

The *in vitro* anticancer activity of Au-Apt-TPE@Zn was firstly examined in SGC-7901 cells by MTT assay. As shown in **Figure 3A**, treatment of cells with Au-Apt-TPE@Zn alone for 24 h showed less cytotoxicity towards SGC-7901 cells. However, Au-Apt-TPE@Zn treatment under 808 nm NIR irradiation (200 mW/cm^2^, 20 min) significantly inhibited SGC-7901 cells growth with a dose-dependent manner. Cells adhesion as an important functional index of cell viability was also detected, and the result showed similar results with that of the MTT assay. As shown in **Figure 3B**, no obvious change of cells adhesion was observed in NIR-treated group and Au-Apt-TPE@Zn-treated group. However, Au-Apt-TPE@Zn treatment under NIR irradiation effectively suppressed the cells adhesion with a time- and dose-dependent manner (**Figure 3B**). The dose-dependent changes of cells morphology further confirmed this growth inhibition effect (**Figure 3C**). These results above all demonstrated that Au-Apt-TPE@Zn under NIR irradiation showed novel photothermal therapy against SGC-7901 cells growth. Moreover, the cells death mechanism induced by Au-Apt-TPE@Zn was also explored by flow cytometry using Annexin-FITC probe. As shown in **Figure 3D**, Au-Apt-TPE@Zn treatment under NIR irradiation dose-dependently induced cells apoptosis in SGC-7901 cells, as convinced by the increase of apoptotic cells from 1.78% (blank) and 5.94% (NIR) to 9.96%, 16.99%, 40.53% and 64.62%, respectively. Taken together, these results indicated that Au-Apt-TPE@Zn under NIR irradiation showed novel photothermal therapy against SGC-7901 cells growth *in vitro* through induction of apoptosis.

### *In vivo* anticancer activity

**Figure 4:**
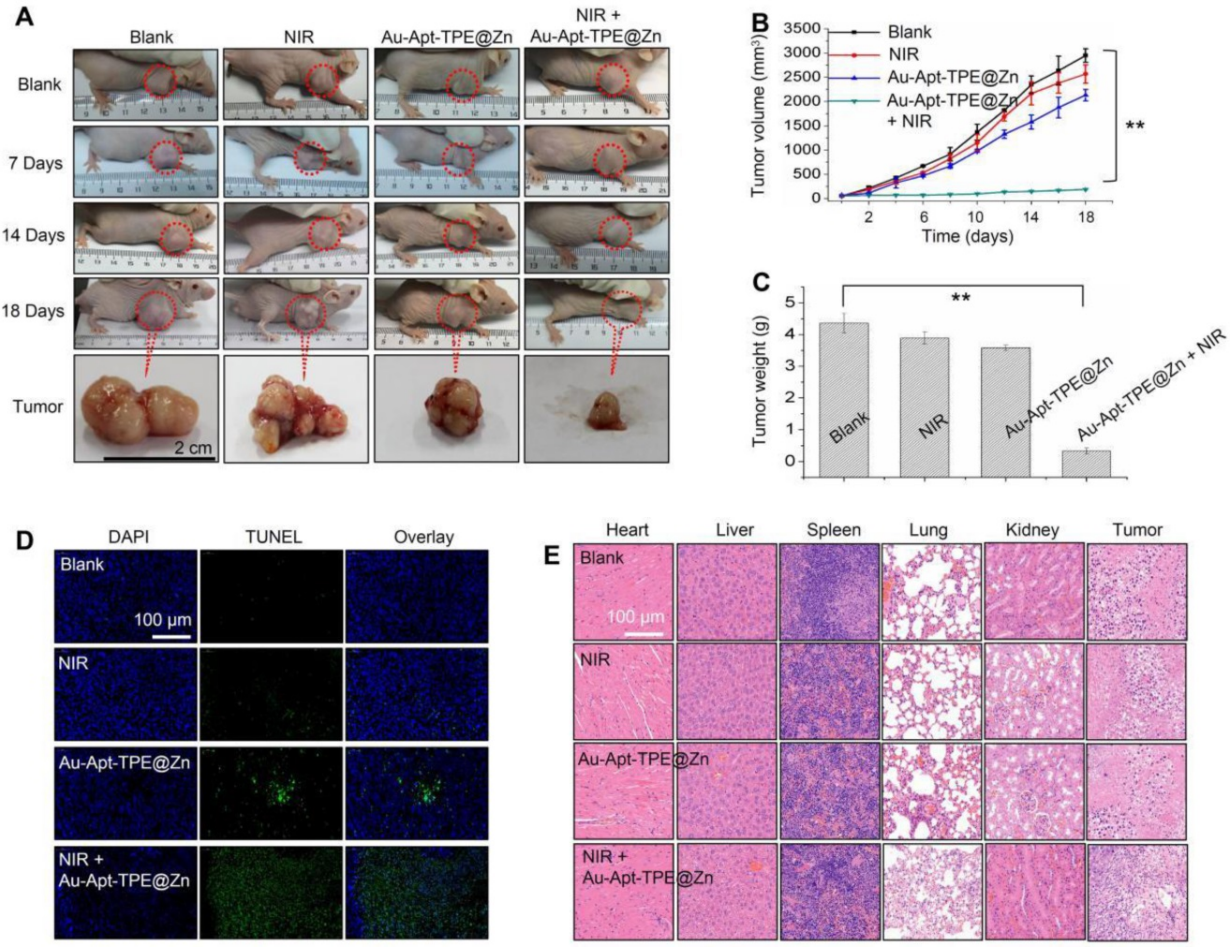
*In vivo* therapeutic effects. (A) Representative images of tumors. Tumors photographs were imaged at 0, 7, 14 and 18 days. Changes of tumor volume (B) and tumor weight (C) measured at 18 days. (D) TUNEL assay for apoptosis* in vivo*. (E) Observation of cells death. Main organs and tumor tissue from mice were collected and analyzed by HE staining for observation of cells death. Data are expressed as means ± SD, **P<0.01 vs. blank group.

The *in vivo* anticancer activity was evaluated in nude mice bearing SGC-7901 human gastric carcinoma xenograft. Representative photos of mice and tumors were arranged in **Figure 4A**, and the results suggested that Au-Apt-TPE@Zn alone slightly inhibited the tumor volume. However, the tumor volume was significantly inhibited after treatment of Au-Apt-TPE@Zn and NIR irradiation. The statistical analysis of tumor volume (**Figure 4B**) and tumor weight (**Figure 4C**) further confirmed this growth inhibition effect in vivo. The in vivo anticancer mechanism by which Au-Apt-TPE@Zn caused cells death was also illuminated by TUNEL assay. As shown in** Figure 4D**, Au-Apt-TPE@Zn alone caused slight cancer cells apoptosis in vivo. Treatment of Au-Apt-TPE@Zn and NIR irradiation resulted in enhanced cancer cells apoptosis in vivo, as indicated by the increased TUNEL-positive cells (green) (**Figure 4D**). The HE staining results also verified that Au-Apt-TPE@Zn treatment with or without local NIR irradiation caused no cells death of mice heart, liver, spleen, lung and kidney, but caused significant cells death of tumor section. Moreover, the mice body weight in different groups showed no drastical fluctuation (**Figure S5**), indicating that the mice were tolerated to treatment and had no acute side effects. Taken together, these results revealed that Au-Apt-TPE@Zn under NIR irradiation showed novel photothermal therapy against SGC-7901 human gastric carcinoma xenograft growth *in vivo* via induction of apoptosis.

### Recognition of early state apoptotic cells

**Figure 5:**
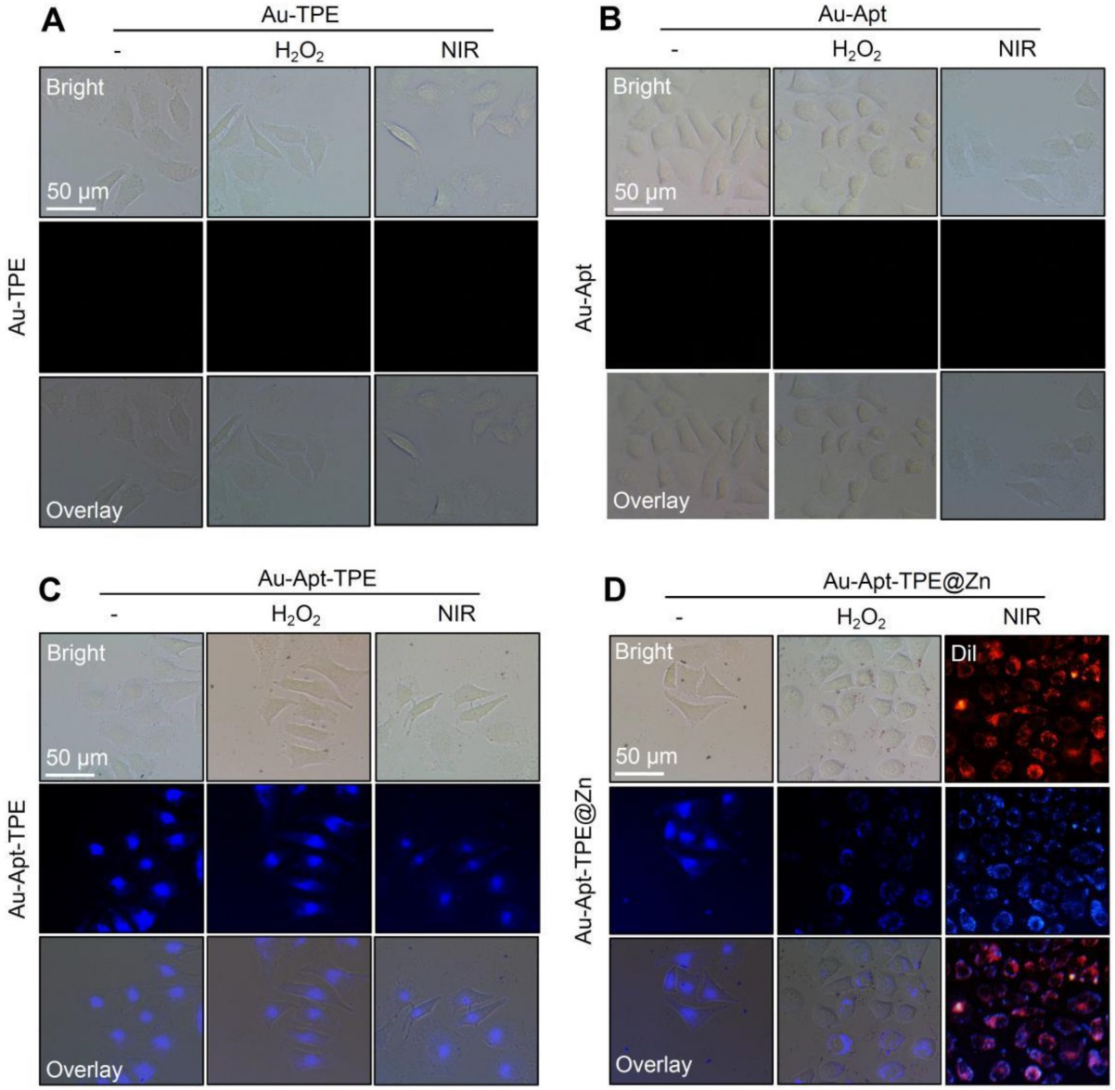
Recognition of early state apoptotic cells. SGC-7901 cells were treated with 250 μM H_2_O_2_ for 6 h and stained with 5 µg/mL Au-TPE (A), 5 µg/mL Au-Apt (B), 5 µg/mL Au-Apt-TPE (C) or 5 µg/mL Au-Apt-TPE@Zn (D) with or without NIR irradiation. The fluorescent images were obtained by fluorescence microscope.

**Figure 6:**
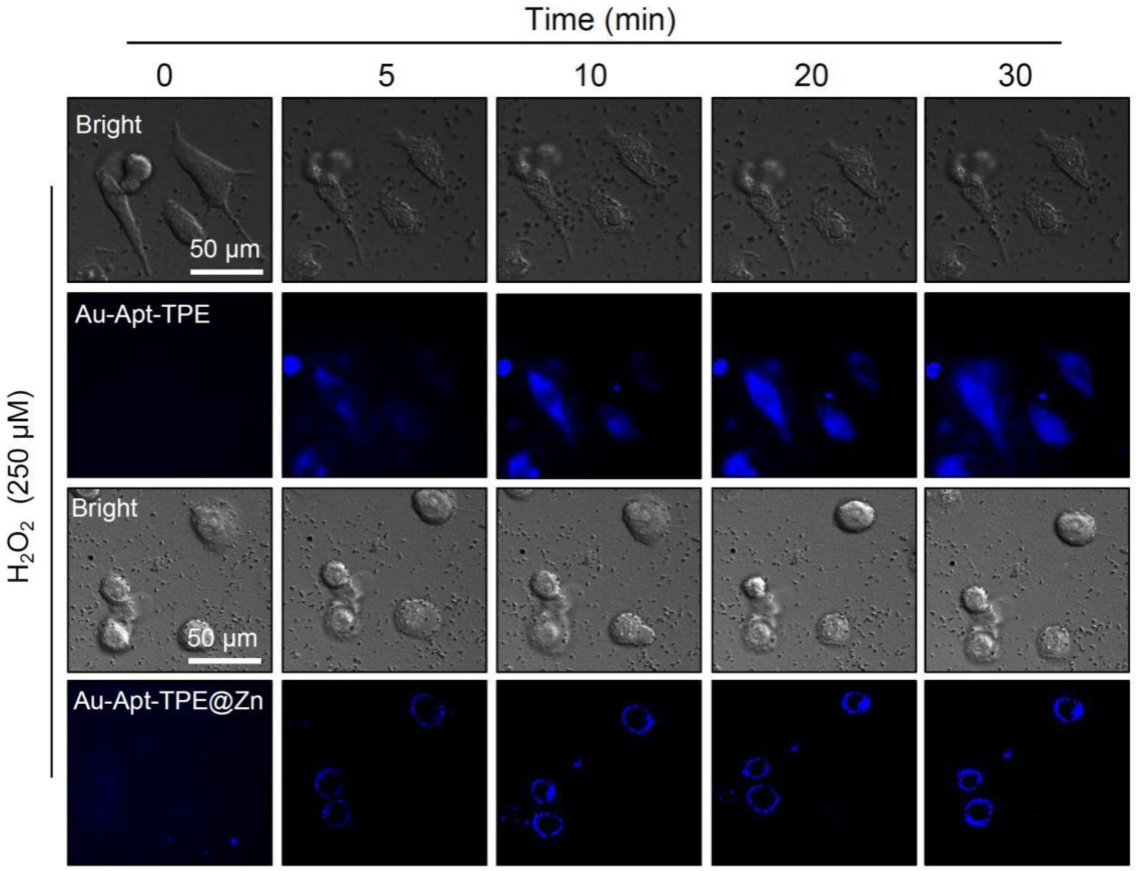
Real-time fluorescence imaging. SGC-7901 cells were treated with 250 μM H_2_O_2_ for 6 h and stained with 5 µg/mL Au-Apt-TPE or 5 µg/mL Au-Apt-TPE@Zn. The fluorescent images were obtained by fluorescence microscope.

The potential of Au-Apt-TPE@Zn to recognize the early state apoptotic cells was evaluated in SGC-7901 cells. Cells were treated with 250 μMH_2_O_2_ for 6 h, which was employed as an early state apoptotic model. As shown in **Figure 5**, H_2_O_2_-treated cells after co-staining with Au-TPE (**Figure 5A**) or Au-Apt (**Figure 5B**) both showed no fluorescence, indicating that Au-TPE or Au-Apt both had no potential to recognize the early state apoptotic cells. H_2_O_2_-treated cells after co-staining with Au-Apt-TPE showed obvious blue fluorescence in nucleus, and the results suggested that Au-Apt-TPE had no ability to recognize the early state apoptotic cells, but validated the AIE characteristics of Au-Apt-TPE by nucleus-targeted aggregation through Apt surface decoration. However, zinc conjunction (TPE@Zn) endowed Au-Apt-TPE@Zn the potential to specifically recognize the apoptotic cells. As shown in **Figure 5D**, H_2_O_2_-treated cells after co-staining with Au-Apt-TPE@Zn showed obvious blue fluorescence in cell membrane, implying that Au-Apt-TPE@Zn had the ability to selectively recognize the membranes of early stage apoptotic cells, but not respond to healthy cells. The real-time fluorescence imaging of Au-Apt-TPE or Au-Apt-TPE@Zn on H_2_O_2_-treated cells further confirmed this specifically recognition (**Figure 6**). Importantly, because of the AIE characteristics of TPE@Zn, Au-Apt-TPE@Zn exhibited low background fluorescence and high signal-to-noise ratio allowing for real-time continuous monitoring of cell apoptosis without washing steps. These findings validated that Au-Apt-TPE@Zn can act as novel fluorescent probe for recognition of early apoptotic cells, which provided valuable diagnosis information on therapeutic efficacy in clinic.

### Photothermal therapeutic efficiency under different NIR irradiation

**Figure 7:**
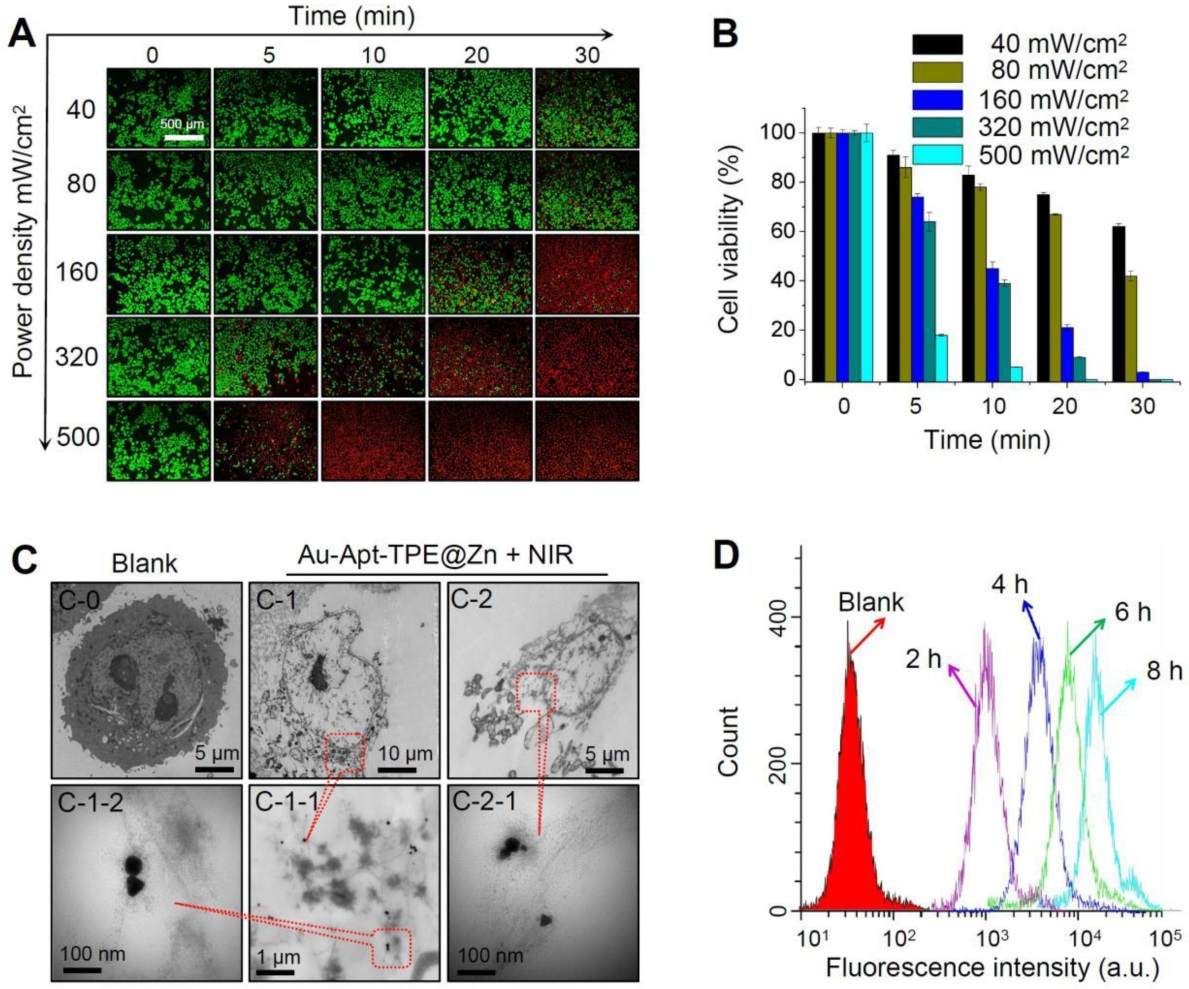
Photothermal therapeutic efficiency and cellular localization. (A) LIVE-DEAD cell assays. SGC-7901 cells were incubated with 30 µg/mL Au-Apt-TPE@Zn for 12 h under different power density of NIR (40, 80, 160, 320, and 500 mW/cm^2^), and the cell death was examined at 0, 5, 10, 20 and 30 min. (B) Cells viability detected by MTT assay. (C) Subcellular localization. TEM was employed to detect the cellular localization of Au-Apt-TPE@Zn. C-0 was the blank group. C-1 and C-2 indicated SGC-7901 cells treated with Au-Apt-TPE@Zn. C-1-1 and C-1-2 were enlarged from C-1. C-2-1 was enlarged from C-2. Red arrows and yellow squares indicated Au-Apt-TPE@Zn NPRs. (D) Flow cytometric profiles of SGC-7901 cells incubation with Au-Apt-TPE@Zn at different times.

Photothermal therapeutic efficiency of Au-Apt-TPE@Zn against SGC-7901 cells growth under different power density of NIR irradiation was detected. LIVE-DEAD assays (Calcine AM and PI co-staining) were firstly employed to elucidate Au-Apt-TPE@Zn-induced cell death. As shown in **Figure 7A**, with the increase of power density and irradiation time, Au-Apt-TPE@Zn under NIR treatment caused significant cells death with a time-and dose-dependent manner, as indicated by the enhanced red fluorescence. Cell viability detected by MTT assay was also conducted to evaluate photothermal therapeutic efficiency. As shown in **Figure 7B**, Au-Apt-TPE@Zn under different dosage of NIR treatment showed enhanced photothermal therapy against SGC-7901 cells growth with a time-and dose-dependent manner. Furthermore, photothermal therapeutic efficiency and cellular localization of Au-Apt-TPE@Zn were examined in subcellular ultrastructure by TEM. As shown in **Figure 7C**, cells after treatment of Au-Apt-TPE@Zn and NIR irradiation were fully disintegrated, and showed unclear and collapsed cell membrane. Meanwhile, the TEM results also ascertained that the subcellular localization of Au-Apt-TPE@Zn was the cell membrane, which convinced the cell membrane-targeted apoptotic recognition and photothermal therapy. Cells death detected by flow cytometry further confirmed the potential photothermal therapeutic efficiency (**Figure 7D**). Taken together, these results indicated that Au-Apt-TPE@Zn under different NIR irradiation treatment displayed varied photothermal therapeutic efficiency.

### Dual-model imaging and *in vivo* biodistribution

**Figure 8:**
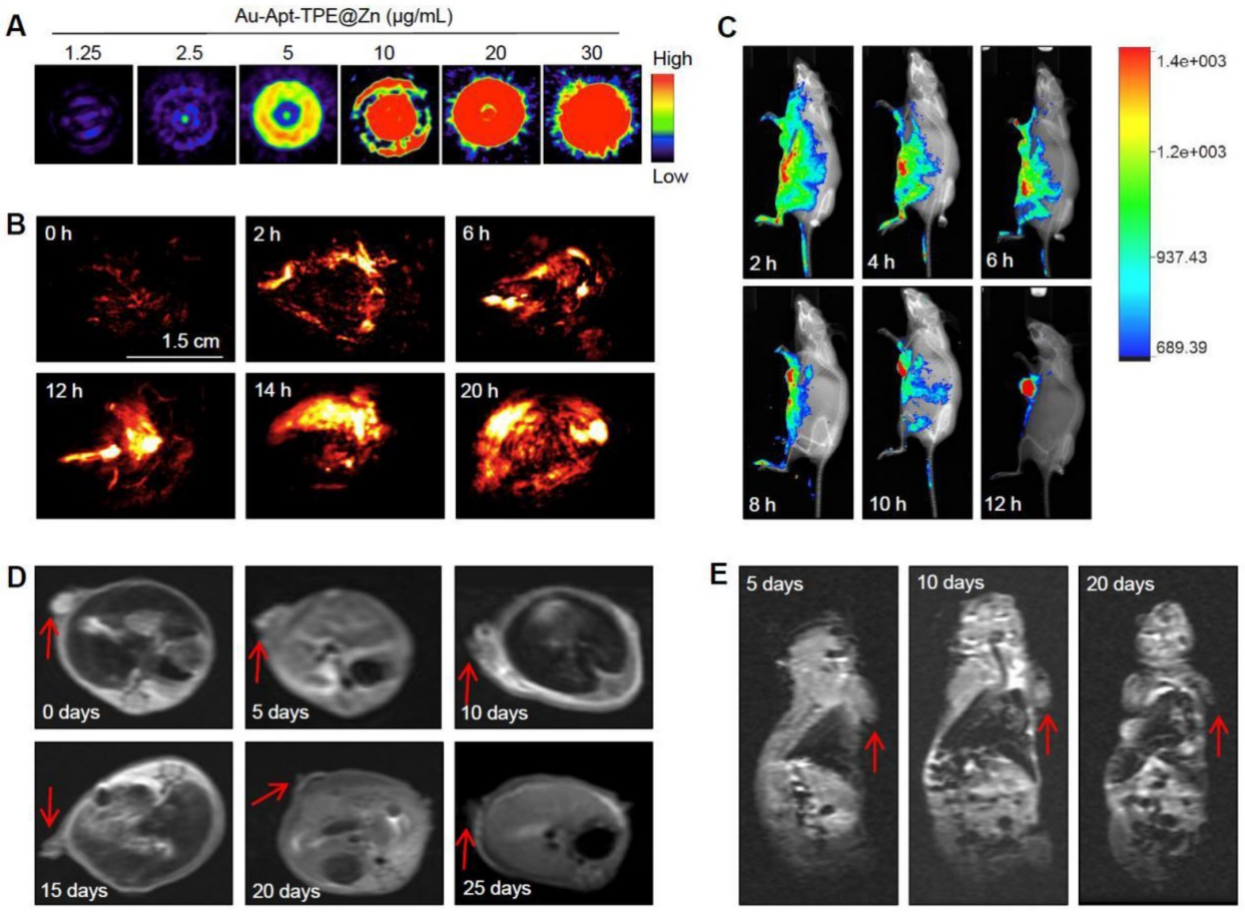
Dual-model imaging and *in vivo* biodistribution. (A) PA signal in vitro. PA signal intensity of Au-Apt-TPE@Zn with different concentrations (1.25, 2.5, 5, 10, 15, 20 and 30 µg/mL) was monitored. (B) Real-time PA images *in vivo*. PA signal of tumor *in vivo* was monitored at different time points. (C) Real-time fluorescence imaging *in vivo*. *In vivo* bioluminescence imaging of the mice was examined at different time points. (D-E) MR images. T_2_-weighted MRI was employed to evaluate the photothermal therapy against SGC-7901 human gastric carcinoma xenograft growth. Red arrows indicated the tumor area. Au-Apt-TPE@Zn (2 mg/kg) was administrated intravenously for all mice.

The dual-model imaging and *in vivo* biodistribution of Au-Apt-TPE@Zn were evaluated in nude mice bearing SGC-7901 human gastric carcinoma xenograft. The photoacoustic (PA) signal of Au-Apt-TPE@Zn under NIR irradiation was firstly monitored *in vitro*. As shown in **Figure 8A**, the PA signal *in vitro* showed continuous enhancement with the increase dosage of Au-Apt-TPE@Zn, indicating the potential application in PA imaging in vivo. The quantitative analysis of photoacoustic signal showed a remarkable positive correlation between the photoacoustic signal and concentration (**Figure S6**), which further highlighted the potential. Secondly, the real-time PA imaging in vivo was also monitored in an animal model. As shown in **Figure 8B**, after administration of Au-Apt-TPE@Zn in vivo, the tumor region of mice under NIR irradiation showed obvious PA signals, which was monitored as early as 2 h, and reached the peak at 10 h. These results suggested that Au-Apt-TPE@Zn mainly accumulated in tumor area, and Au-Apt-TPE@Zn under NIR irradiation can be applied for *in vitro* and *in vivo* PA imaging with high spatial resolution advantage. The *in vivo* biodistribution and imaging of Au-Apt-TPE@Zn was also detected by a real-time biofluorescent method. As shown in **Figure 8C**, after administration of Au-Apt-TPE@Zn in vivo, the mice under NIR irradiation showed notable biofluorescence in tumor and surrounding region. The fluorescent signals were detected in the tumor region as early as 6 h, and eventually achieved a complete tumor-targeted aggregation at 12 h, which confirmed the cancer-targeted imaging of Au-Apt-TPE@Zn in vivo. Magnetic resonance imaging (MRI) was accepted as effective tool for examining tissue lesions. Damaged tissue, such as bleeding or edema, usually showed enhanced MR signal. Herein, a dynamic T_2_-weighted MRI was employed to evaluate the photothermal therapy against SGC-7901 human gastric carcinoma xenograft growth. As shown in **Figure 8D and E**, a large white area (T_2_-weighted signal) can be found in tumor region of blank group. However, the white area gradually became darker and smaller with a time-dependent manner. The MRI results intuitively indicated the effective photothermal therapy against tumor growth *in vivo*. Taken together, these results above all validated that our design of using Au nanoprism with AIE materials and dual-targeted decoration could be an effective strategy in cancer dual-model imaging and precise cancer photothermal therapy.

### Molecular mechanism induced by Au-Apt-TPE@Zn

**Figure 9:**
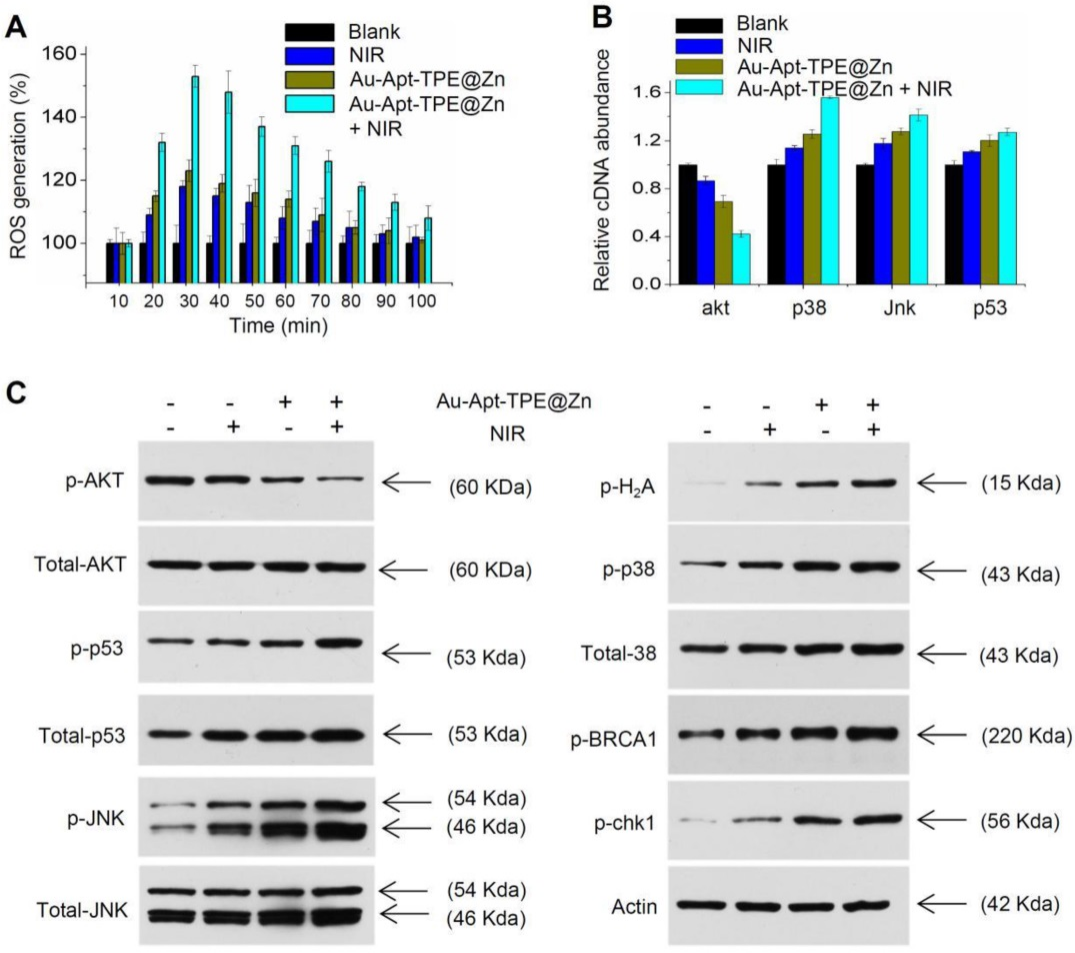
Molecular mechanism induced by Au-Apt-TPE@Zn. (A) Real-time ROS regeneration. ROS generation was detected by DCFH-DA probe as described in method section. (B) Relative cDNA abundance. The relative cDNA abundance of akt, p38, Jnk and p53 were detected by real-time RT-PCR. (C) Western blotting for protein expression. MAPKs, PI3K/AKT and DNA damage signal molecular were examined by western blotting method. Data are means ± SD, * *P<*0.05, ** *P<* 0.01, vs. blank group.

Photothermal therapy may cause generation of cytotoxic ROS. ROS as up-stream active molecular can damage DNA, proteins and lipids, and eventually cause cancer cells apoptosis, which was accepted as the main anticancer mechanism of photothermal therapy 8, 14. Hence, the real-time course of ROS generation in Au-Apt-TPE@Zn-treated cells with or with NIR irradiation was detected. As shown in **Figure 9A**, cells treated with NIR or Au-Apt-TPE@Zn alone showed light ROS increase. However, Au-Apt-TPE@Zn under NIR irradiation caused enhanced ROS generation detected as early as 20 min, and reached the peak at 30 min. The result indicated that Au-Apt-TPE@Zn under NIR irradiation caused ROS overproduction. Subsequently, down-stream molecular signaling pathway was explored in Au-Apt-TPE@Zn-treated SGC-7901 cells with or with NIR irradiation. As shown in **Figure 9B**, the relative cDNA abundance of AKT, p38, JNK and p53 were detected by RT-PCR, and the results showed that NIR irradiation significantly increased the cDNA abundance of p38, jnk and p53, but decreased the cDNA abundance of akt in Au-Apt-TPE@Zn-treated cells. Moreover, several molecular signals were examined in protein level. As shown in **Figure 9C**, treatment of cells with NIR or Au-Apt-TPE@Zn alone only caused slight changes of p-AKT, p-p53, p-JNK, p-p38, p-BRCA1 and p-chk1. However, Au-Apt-TPE@Zn under NIR irradiation caused enhanced dysfunction of p-AKT, p-p53, p-JNK, p-p38, p-BRCA1 and p-chk1. Taken together, these results demonstrated that ROS overproduction, dysfunction of MAPKs, PI3K/AKT and DNA damage all contributed to Au-Apt-TPE@Zn-mediated photothermal therapy.

### Safety evaluation* in vitro* and* in vivo*

**Figure 10:**
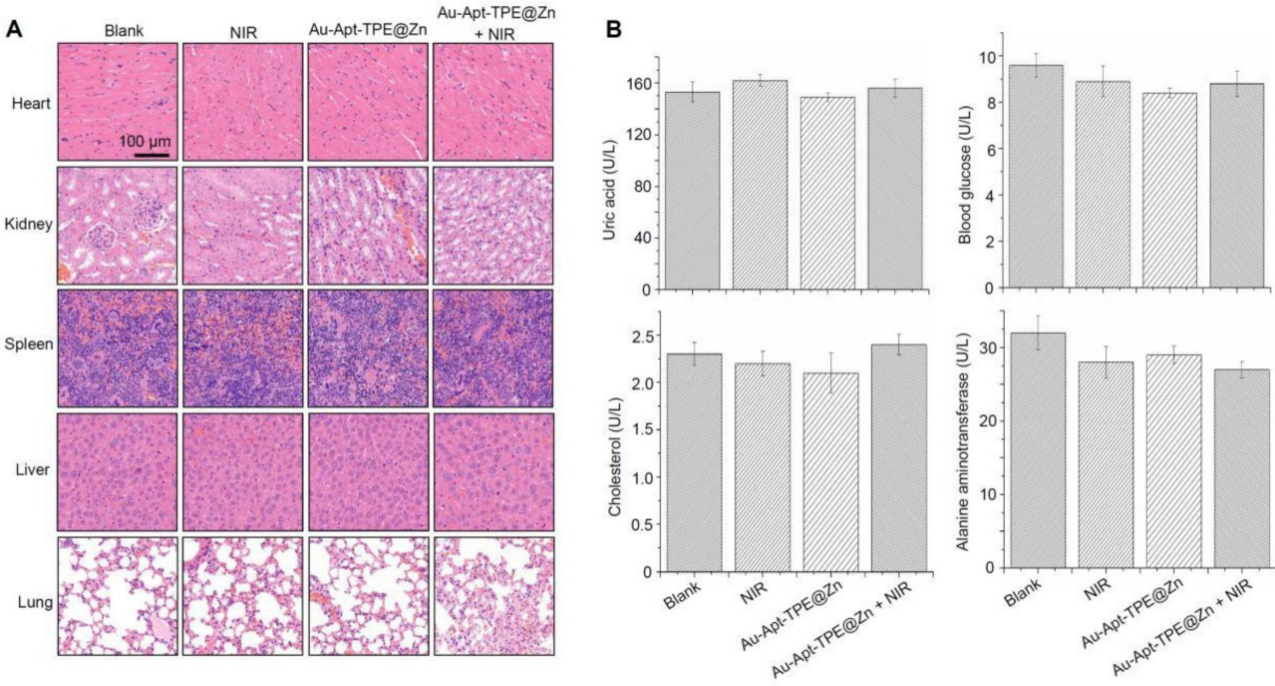
Safety evaluation. (A) Histopathological changes. Histopathological changes in main organs was examined by HE staining. (B) Blood biochemistry. Liver function markers, kidney function markers, blood glucose, and blood lipids were all detected.

Side effect and biocompatibility of the Au-Apt-TPE@Zn were examined to evaluate its safety. Au-Apt-TPE@Zn in physiological condition showed stable size (**Figure S7**), and Au-Apt-TPE@Zn *in vivo* after metabolism by mice remained stable morphology (**Figure S8**), indicating the good stability and biocompatibility *in vitro* and *in vivo*. H&E staining was performed for histopathological changes. As shown in the **Figure 10**A, the NIR, Au-Apt-TPE@Zn, and Au-Apt-TPE@Zn+NIR treatment groups all showed similar histological features compared with the blank groups, and no obvious impairment or inflammation was observed in all groups. Blood biochemical assays were also carried out to examine possible changes in the blood biochemistry of mice after treatment. As shown in **Figure 10B**, the levels of cholesterol, blood glucose, uric acid, and alanine aminotransferase (index for function of liver, kidney, blood glucose, and blood lipids, respectively) all showed no significant changes comparing to that of blank group. Other hematological parameters of mice treated with Au-Apt-TPE@Zn or/and NIR were also detected, and the results indicated that mice treated with 4 mg/kg Au-Apt-TPE@Zn and NIR showed slight changes of RBC, WBC, MCV, HCT, HGB, MCH, AST and ALT (**Table S2**). Mice treated with 1 and 2 mg/kg of Au-Apt-TPE@Zn with and/or NIR showed no significant changes of hematological parameters (**Table S2**). Moreover, macrophage toxicity in vitro and in vivo was also examined, and the result showed that treatment with Au-Apt-TPE@Zn and NIR displayed significant macrophage toxicity in vitro with a dose-dependent manner. However, only 4 mg/kg of Au-Apt-TPE@Zn and NIR showed slight macrophage toxicity *in vivo* against kupffer cells in liver (**Figure S9**). Au-Apt-TPE@Zn (1 and 2 mg/kg) with or without NIR showed no toxicity towards kupffer cells (**Figure S9**). Taken together, these results all indicated the less side effects and high biocompatibility of Au-Apt-TPE@Zn with potential application in clinic.

## Conclusion

**Figure 11:**
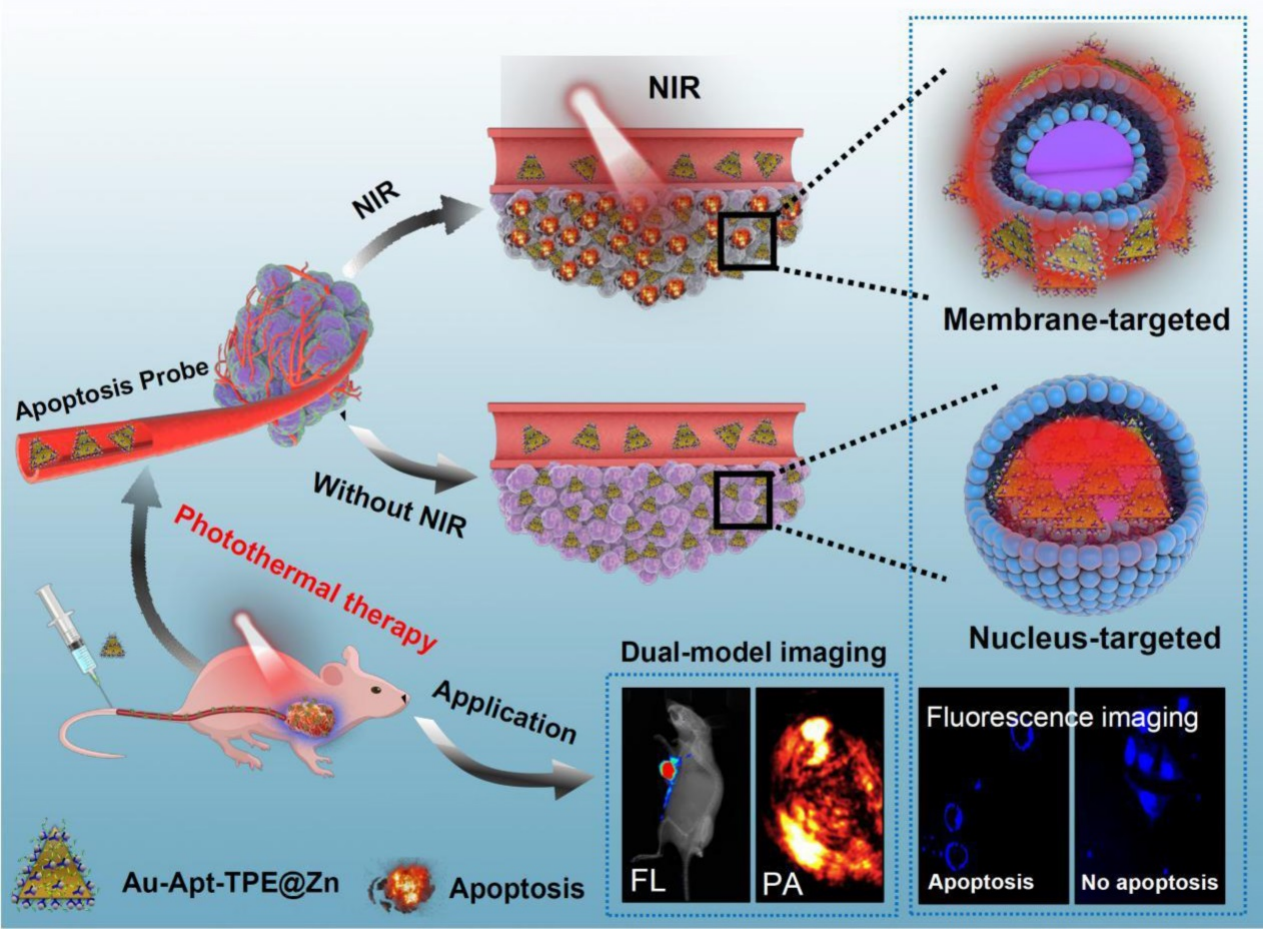
Design of multifunctional Au nanoprism (Au-Apt-TPE@Zn) with AIE materials and dual-targeted decoration for recognition of early apoptosis, dual-model imaging (cancer-targeted fluorescence imaging and light-up photoacoustic imaging) and precise cancer photothermal therapy.

In this study, gold nanoprism was prepared by microwave one-step synthesis, and a multifunctional gold nanoprism (Au-Apt-TPE@Zn) with good monodispersity, size stability and consistent spectral characteristics was obtained by surface decoration of DNA aptamer (Apt) and Zinc-tetraphenylethene (TPE@Zn). TPE synthesized here showed aggregation-induced emission (AIE) characteristics, and zinc conjunction (TPE@Zn) endowed Au-Apt-TPE@Zn with the cell membrane-targeted ability to selectively recognize the membranes of early stage apoptotic cells but not respond to healthy cells, which provided valuable diagnosis information on therapeutic efficacy. Au-Apt-TPE@Zn achieved specifically nuclear-targeted ability by surface decoration of AS1411 DNA aptamer. Au-Apt-TPE@Zn under NIR irradiation showed strong photothermal therapy against SGC-7901 human gastric carcinoma cells growth *in vitro* and *in vivo* by induction of cells apoptosis and regulating multiple signal crosstalk. Au-Apt-TPE@Zn *in vivo* under NIR irradiation showed deep penetration and cancer-targeted fluorescence imaging and light-up photoacoustic imaging properties. Meanwhile, Au-Apt-TPE@Zn under NIR irradiation exhibited less side effect and high biocompatibility. Our findings validated the design of using Au nanoprism with AIE materials and dual-targeted decoration could be an effective strategy in recognition of early apoptosis, dual-model imaging and precise cancer photothermal therapy (**Figure 11**).

## Supplementary Material

Supplementary figures and tables.
