# Dual-Targeted Gold Nanoprism for Recognition of Early Apoptosis, Dual-Model Imaging and Precise Cancer Photothermal Therapy: Erratum

**DOI:** 10.7150/thno.85592

**Published:** 2023-05-15

**Authors:** Weiwei Zhang, Xiaoyuan Ding, Hao Cheng, Chenyang Yin, Jing Yan, Zhipeng Mou, Weiyun Wang, Danxi Cui, Cundong Fan, Dongdong Sun

**Affiliations:** 1 School of Life Sciences, Anhui Agricultural University, Hefei, 230036, China; 2 Key Lab of Cerebral Microcirculation in Universities of Shandong, Shandong First Medical University & Shandong Academy of Medical Sciences, Taian, Shandong, 271000, China

The authors regret that this article contains an error in Figure 7A. The original version of the article contained an incorrect image in Figure 7A where the power density: 40 mW/cm^2^ + 30 min group and the power density: 80 mW/cm^2^ + 30 min group were inadvertently duplicated. This experiment was easily to be repeated, but generated 25 groups, which made two images inadvertently duplicated. The authors show regret over this mistake. The correct version of Figure 7A after repeating the experiment is shown below.

The correction made in this erratum does not affect the original data and conclusions. The authors apologize for any inconvenience that the errors may have caused.

## Figures and Tables

**Figure 7A:**
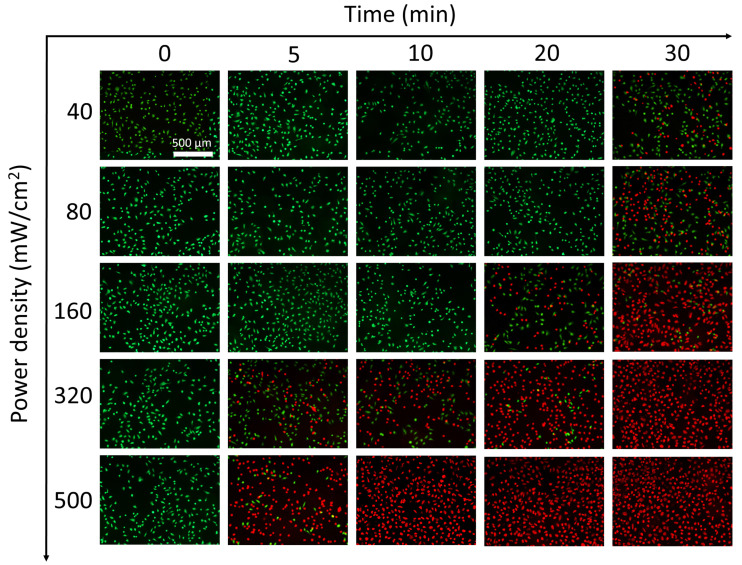
Corrected figure for original Figure 7A.

