# Supplementary material for: Dual-Targeted Gold Nanoprism for Recognition of Early Apoptosis, Dual-Model Imaging and Precise Cancer Photothermal Therapy
Source: Theranostics. 2019 Jul 28;9(19):5610–25. doi: 10.7150/thno.34755 (PMC6735394; doi:10.7150/thno.34755)
Supplement: Supplementary file 1 — Supplementary figures and tables. [file thnov09p5610s1.pdf]

Supporting Information

for

Dual-Targeted Gold Nanoprism for Recognition of Early Apoptosis, Dual-Model Imaging and Precise Cancer Photothermal Therapy

Weiwei Zhang[#,a], Xiaoyuan Ding[#,a], Hao Cheng[a], Chenyang Yin[a], Jing Yan[a], Zhipeng Mou[a], Weiyun Wang[a], Danxi Cui[a], Cundong Fan[*b], Dongdong Sun[*a]

[a] School of Life Sciences, Anhui Agricultural University, Hefei, 230036, China

[b] Key Lab of Cerebral Microcirculation in Universities of Shandong, Shandong First Medical University & Shandong Academy of Medical Sciences, Taian, Shandong, 271000, China

[#]The authors contributed equally to this work.

***Corresponding author:**

Dongdong Sun, School of Life Sciences, Anhui Agricultural University, Hefei, 230036, China. Tel: +86-0551-65786703; fax: +86-0551-65786703. Email: sunddwj@ahau.edu.cn

Cundong Fan, 2 Yingsheng East Road, Shandong First Medical University & Shandong Academy of Medical Sciences, Taian, Shandong, 271000, China. Email: cdfan@tsmc.edu.cn. Tel: +86-0538-6230027; fax: +86-0538-6230027

Methods

1.1 Calculation of photothermal conversion efficacy. The photothermal conversion efficacy of Au-Apt-TPE@Zn NPRs was calculated using the following equation.

$$\eta = (hS\Delta T_{max}-Q_s)/I(1-10^{-A})$$

$$\tau_s = m_D C_D/hS$$

Where h is heat transfer coefficient, S is the surface area, ΔT_{max} is the equilibrium temperature, Ts is the ambient temperature of the surroundings, Q_s express heat absorbed by the cell, I is the laser power, and A is the absorbance of Au-Apt-TPE@Zn NPRs at 808 nm. Where τ_s is the a sample system time constant, m_{H2O} is the weight of water, and C_{H2O} is the specific heat capacity of water.

1.2 Stability of the Au-Apt-TPE@Zn NPRs *in Vivo.* First, the Au-Apt-TPE@Zn NPRs (2 mL, with a dose of 2 mg/kg material) were injected into the mice via the tail vein. Second, its urine and feces were carefully collected within 24 h. Third, to fully break up the feces, a 30 min ultrasonic process was conducted. Then, the obtained products were centrifuged by a low speed (8000 rpm, 10 min) for the remove of bulky impurities. The same centrifugation processes were performed for urine. Fourth, both the treated feces and urine were dialyzed for 48 h, respectively. Finally, the purified urine and feces were characterized by TEM technique.

Results

Table S1. Photothermal conversion efficacy of Au-Apt-TPE@Zn NPRs comparing with other types of gold nanostructures.

Gold nanomaterials	Photothermal conversion efficacy	Refercence
Gold nanoprisms	67.2%	This study
Gold nanoshells	13%	[53]
Gold nanords	21%	[54]
Gold hexapods	29.6%	[55]
Gold nanocages	63%	[55]
Gold nanoprisms	69.3%	[52]
Gold bellflowers	74%	[55]

52. Ma X, Cheng Y, Huang Y, Tian Y, Wang S, Chen Y: PEGylated gold nanoprisms for photothermal therapy at low laser power density. *RSC Advances*, 5(99), 81682-81688 (2015).

53. Hessel CM, Pattani VP, Rasch M *et al.*: Copper selenide nanocrystals for photothermal therapy. *Nano Lett*, 11(6), 2560-2566 (2011).

54. Huang P, Lin J, Li W *et al.*: Biodegradable gold nanovesicles with an ultrastrong plasmonic coupling effect for photoacoustic imaging and photothermal therapy. *Angew Chem Int Ed Engl*, 52(52), 13958-13964 (2013).

55. Zeng J, Goldfeld D, Xia Y: A plasmon-assisted optofluidic (PAOF) system for measuring the photothermal conversion efficiencies of gold nanostructures and controlling an electrical switch. *Angew Chem Int Ed Engl*, 52(15), 4169-4173 (2013).

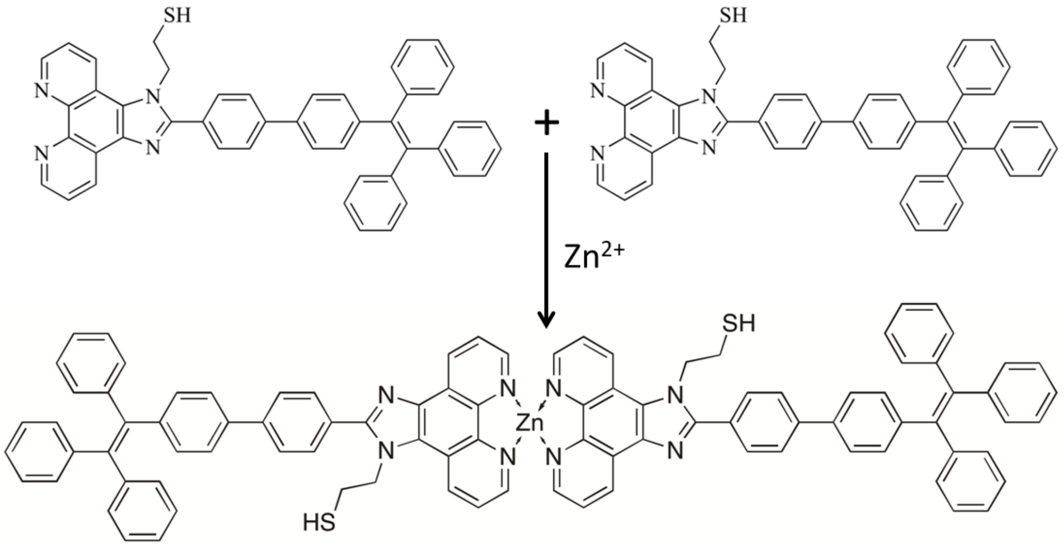

Figure S1. The synthetic route of TPE@Zn. An aqueous solution of zinc perchlorate hexahydrate (10 mL, 10 mmol) were added to Au-Apt-TPE NPRs solution (10 mL) and stirred for 2 h at room temperature.

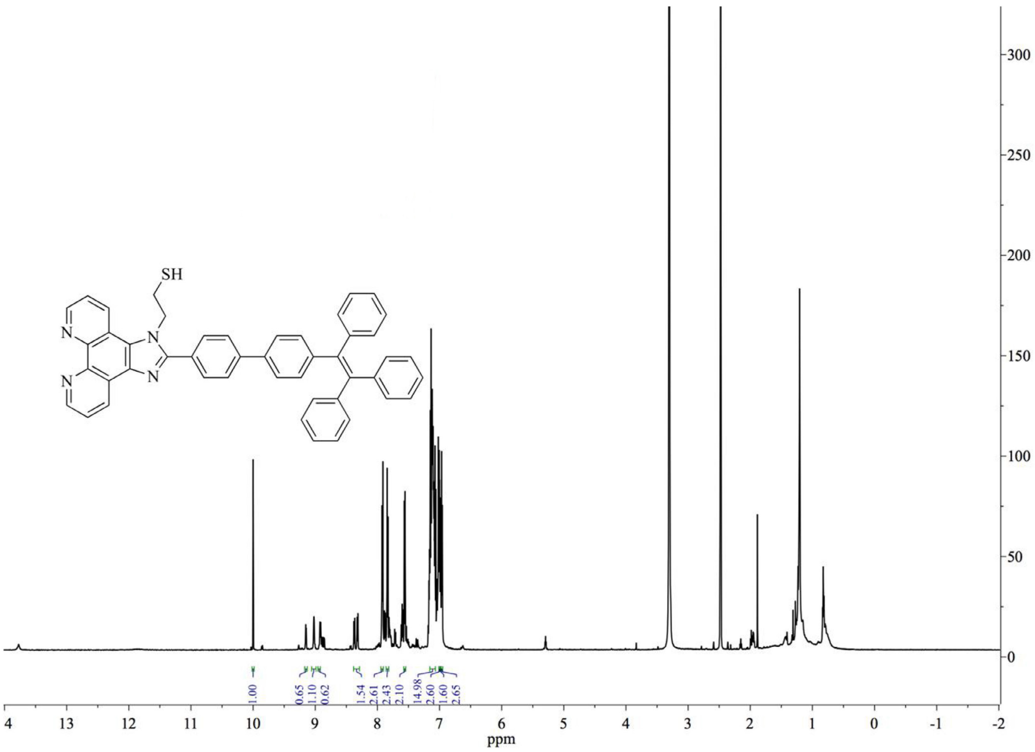

Figure S2. ^{1}H NMR of the TPE-SH in DMSO-d6 (^{1}H NMR (600 MHz, DMSO-d6) δ 10.00 (s, 1H), 9.02 (d, J = 4.1 Hz, 1H), 8.92 (s, 1H), 8.32 (d, J = 8.1 Hz, 2H), 7.92 (dd, J = 8.4, 3.7 Hz, 3H), 7.84 (d, J = 8.2 Hz, 2H), 7.56 (d, J = 8.3 Hz, 2H), 7.16 – 7.07 (m, 15H), 7.01 – 6.99 (m, 3H), 6.99 (q, J = 2.5, 2.0 Hz, 2H), 6.96 (dd, J = 8.1, 1.5 Hz, 3H).).

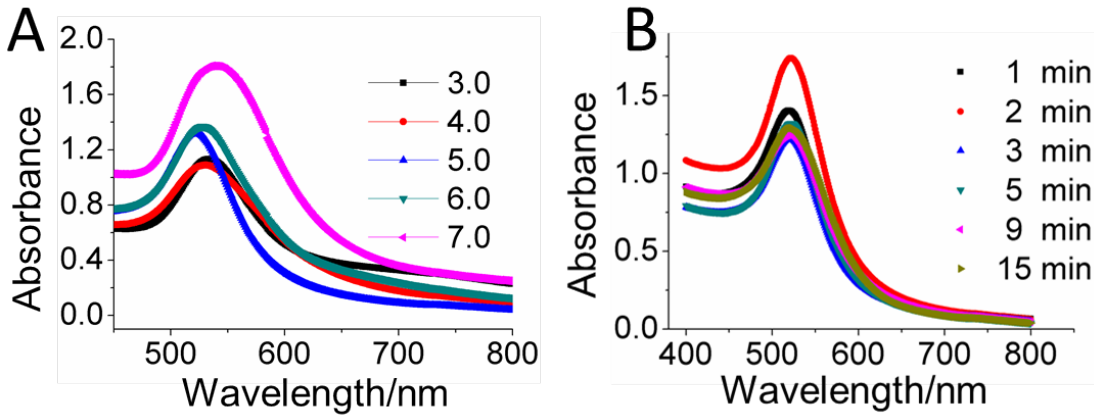

Figure S3. Absorption spectra of different synthesis conditions of Au NPRs. (A) Different pH from 3.0 to 7.0; (B) different microwave times.

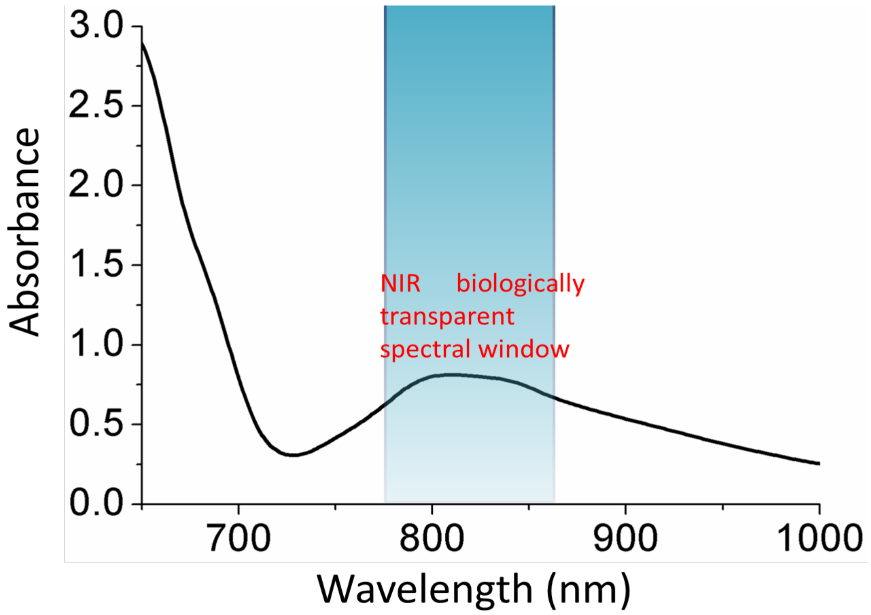

Figure S4. The extinction spectrum of Au-Apt-TPE@Zn shows a localized surface plasmon resonance centered at 808 nm, which is within the biologically transparent spectral window. This strong absorption at NIR wavelengths ensures that light can penetrate several mm into tissue.

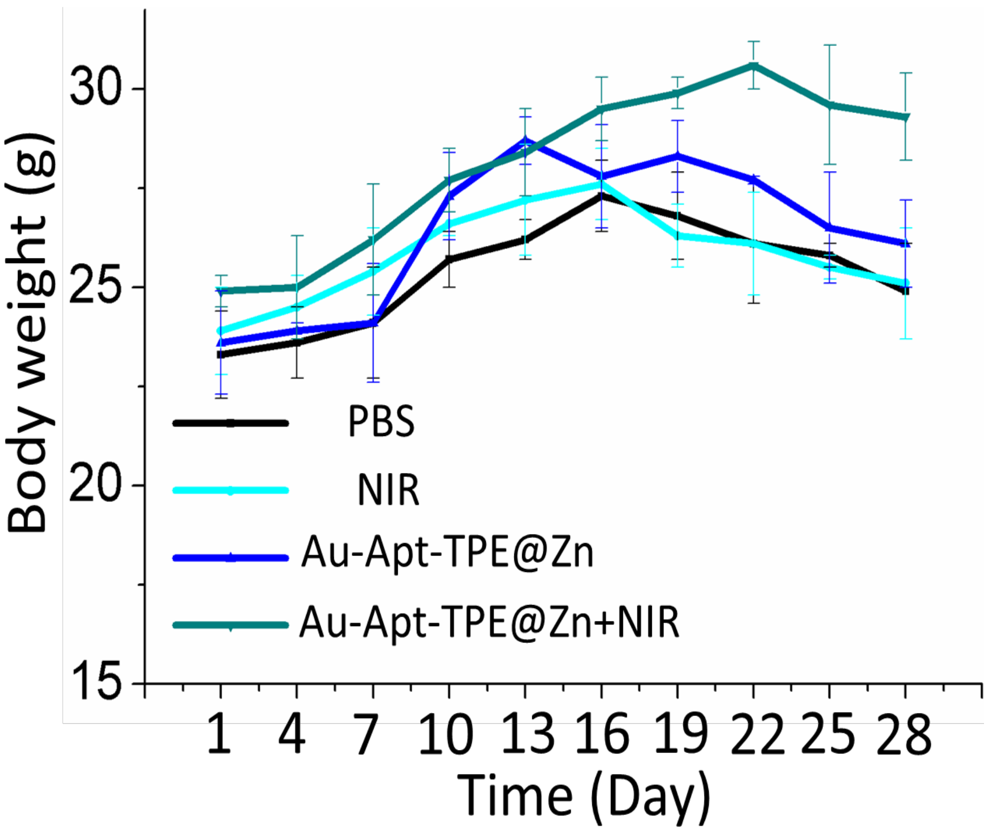

Figure S5. Body weight changes of different groups on the SGC-7901-bearing mice. A dosage of 2 mg/kg was administrated intravenously for all mice. The concentration of PBS was 100 μM.

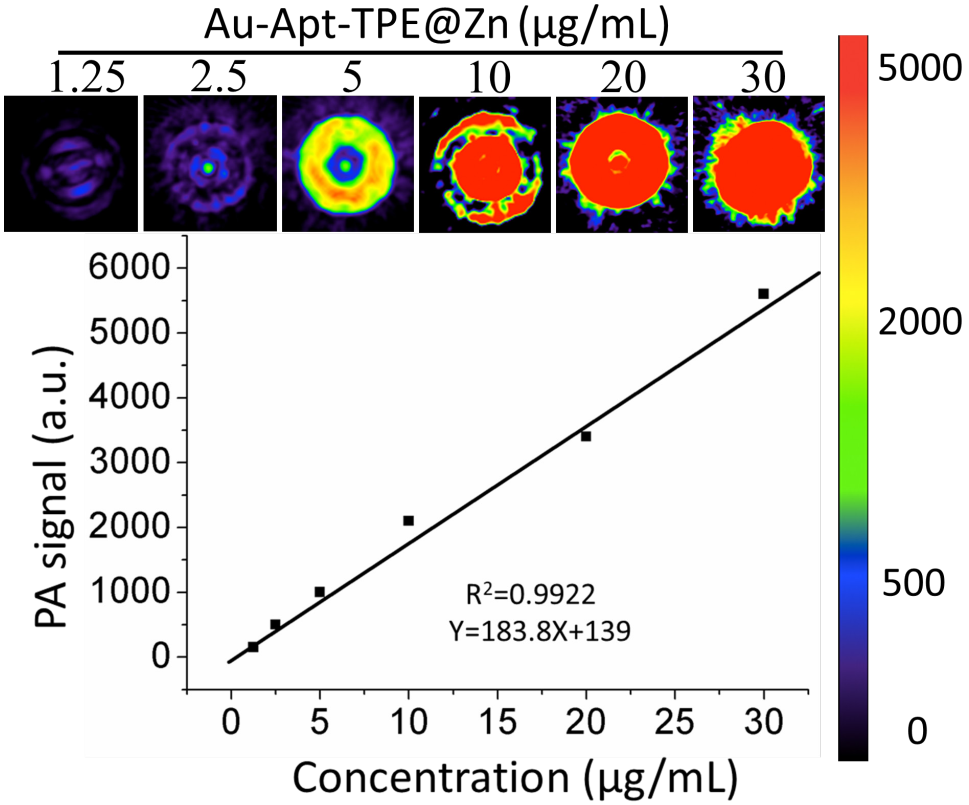

Figure S6. PA images and PA intensity of the Au-Apt-TPE@Zn NPRs aqueous dispersions at different concentrations.

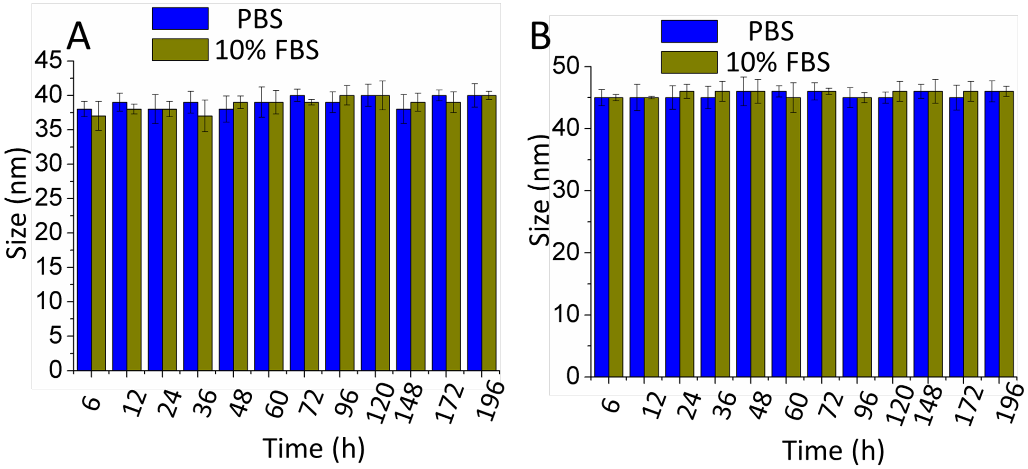

Figure S7. Stability of different nanomaterial. (A) Changes in the hydrodynamic size of Au NPRs in PBS (100 μM) and 10% FBS. (B) Changes in the hydrodynamic size of Au-Apt-TPE@Zn in PBS (100 μM), and 10% FBS.

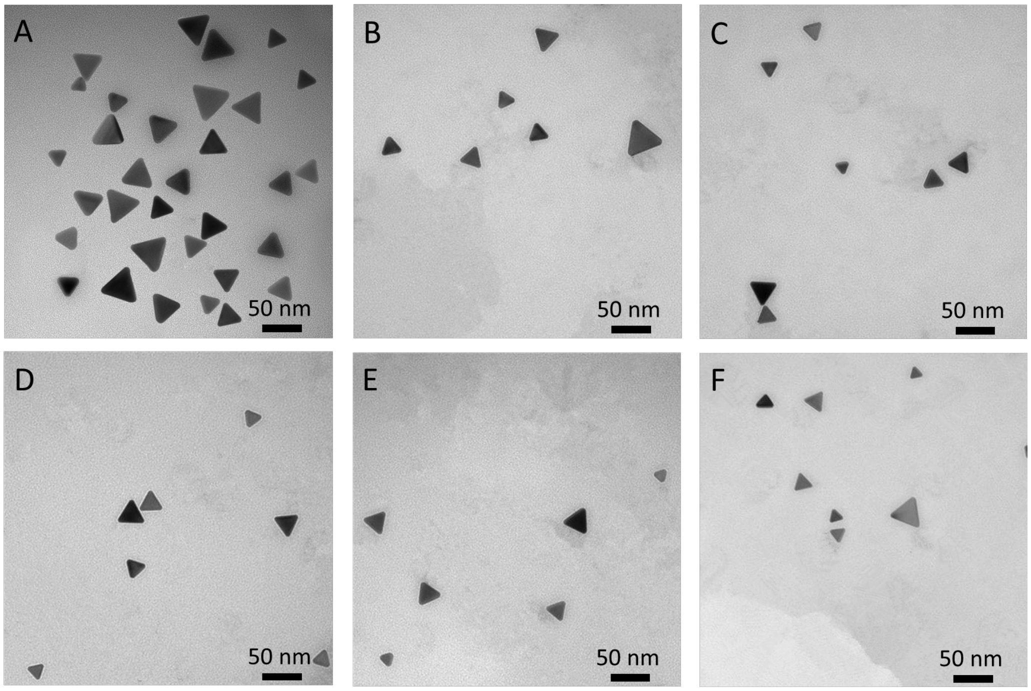

Figure S8. The comparison of the Au-Apt-TPE@Zn NPRs before (A) and after metabolism by mice (B, C: the samples from urine; D, E, F: the samples from feces). Both urine and feces were collected at the period of time from tail vein injection to 24 h. The Au-Apt-TPE@Zn NPRs could well keep their morphology *via* metabolism processes, which indicated that the Au-Apt-TPE@Zn NPRs were enough robust for *in vivo* applications. A dosage of 2 mg/kg was administrated intravenously for all mice.

Table S2. Hematological parameters of mice treated with Au-Apt-TPE@Zn or/and NIR.

Parameter	Unit	Blank	Tumor	NIR	Au-Apt-TPE@Zn (mg/kg)			NIR + Au-Apt-TPE@Zn (mg/kg)		
					1	2	4	1	2	4
RBC	10^{12}/L	8.45 ± 0.38	8.26±0.24	8.01±0.22	7.98±0.12	7.79±0.35	7.27±0.21	7.63±0.23	7.42±0.09	6.89±.0.34*
WBC	10^{9}/L	10.98 ± 0.45	10.23±0.46	10.12±0.36	9.89±0.55	9.28±0.62	8.64±0.26*	9.28±0.12	8.86±0.31	8.15±0.37*
MCV	fL	66.33 ± 1.28	67.38±1.62	67.85±1.25	67.95±1.16	68.24±1.56	68.67±2.13	68.01±1.61	68.92±2.01	69.44±1.42*
HCT	%	56.04 ± 1.85	55.65±1.51	54.34±1.47	54.22±1.24	53.15±1.96	49.92±1.23*	51.89±1.16	51.13±1.38	47.84±1.41**
HGB	g/L	116.10 ± 7.51	115.26±6.65	115.12±7.54	114.32±5.34	112.55±5.18	109.84±5.88*	112.31±5.27	110.28±7.23	107.65±6.87*
MCH	pg	13.73 ± 0.44	13.95±0.99	14.37±0.34	14.32±0.25	14.44±0.26	15.11±0.65	14.72±0.28	14.86±0.69	15.62±0.92
AST	U/L	116.34 ± 8.69	119.32±9.17	121.54±8.12*	118.54±8.37	120.55±8.25	124.65±9.76*	121.45±9.18	123.51±7.52	130.06±6.22*
ALT	U/L	46.57 ± 2.26	49.25±2.04	48.31±2.17	47.34±1.97	47.49±2.68	48.86±1.87	48.35±2.55	49.25±1.98	50.25±2.64*

RBC number of red blood cells, WBC number of white blood cells, MCV mean corpuscular volume, HCT hematocrit, HGB hemoglobin, MCH mean corpuscular hemoglobin, AST aspartate aminotransferase, ALT alanine aminotransferase. Value are expressed as mean ± SD for mice in each group (n=5). BALB/c male nude mice bearing SGC-7901 cells were treated with Au-Apt-TPE@Zn or/and NIR according to the method section. Blood samples were collected and the hematological parameters of all groups mice were analyzed by ACL-200 blood autoanalyzer (Beckman Coulter Corp., USA). "*" indicates a significant difference from blank group (P<0.05); "**" indicates a significant difference from blank group (P<0.01).

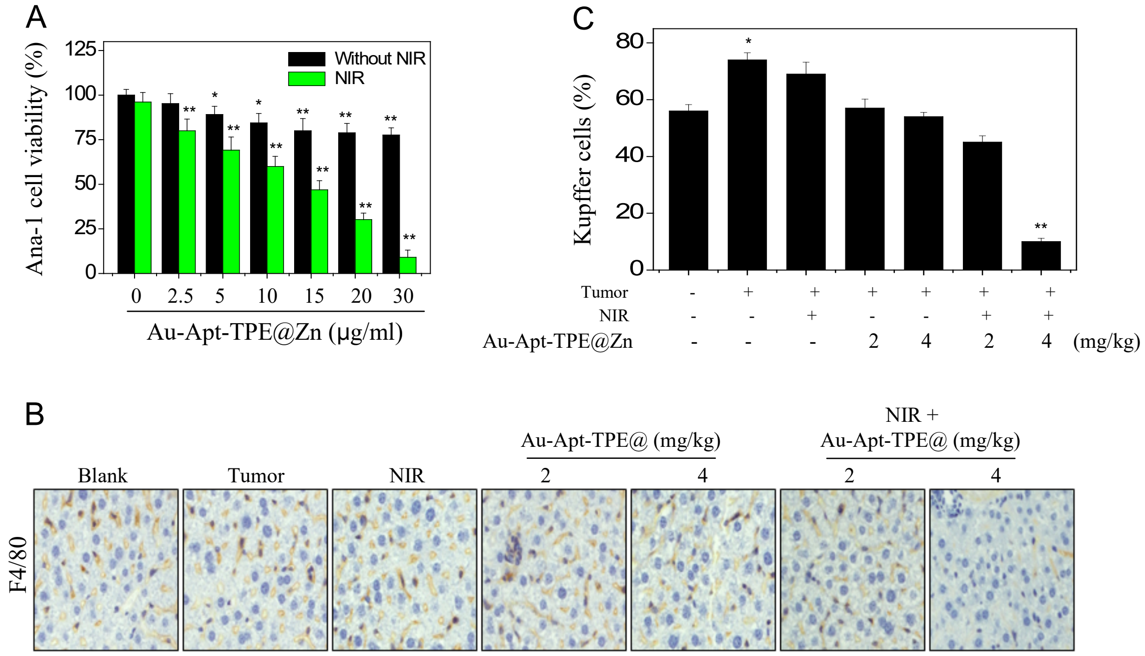

Figure S9. Macrophage toxicity in vitro and in vivo. (A) Macrophage toxicity in vitro. Ana-1 mice macrophages (5×10^4 cells/well) seeded in 96-wells plate were exposed to 2.5-30 μg/ml Au-Apt-TPE@Zn for 12 h with or without NIR treatment (200 mW/cm^2, 20 min). Cells viability was detected by MTT assay. (B) Macrophage toxicity in vivo. Nude mice were exposed to 2 and 4 mg/kg Au-Apt-TPE@Zn with or without NIR treatment (808 nm, 320 mW/cm^2, 20 min). Kupffer cells in liver were immunostained with anti-F4/80 antibody. (C) Statistic analysis of kupffer cells. F4/80^+ cells were calculated by manual counting. *P<0.05 vs. blank, **P<0.01 vs. blank.
